# Integrated Network Analysis to Identify Key Modules and Potential Hub Genes Involved in Bovine Respiratory Disease: A Systems Biology Approach

**DOI:** 10.3389/fgene.2021.753839

**Published:** 2021-10-18

**Authors:** Aliakbar Hasankhani, Abolfazl Bahrami, Negin Sheybani, Farhang Fatehi, Roxana Abadeh, Hamid Ghaem Maghami Farahani, Mohammad Reza Bahreini Behzadi, Ghazaleh Javanmard, Sadegh Isapour, Hosein Khadem, Herman W. Barkema

**Affiliations:** ^1^ Department of Animal Science, College of Agriculture and Natural Resources, University of Tehran, Karaj, Iran; ^2^ Nuclear Agriculture Research School, Nuclear Science and Technology Research Institute, Karaj, Iran; ^3^ Department of Animal and Poultry Science, College of Aburaihan, University of Tehran, Tehran, Iran; ^4^ Department of Animal Science, Science and Research Branch, Islamic Azad University, Tehran, Iran; ^5^ Department of Animal Science, Faculty of Agriculture, Yasouj University, Yasouj, Iran; ^6^ Department of Agronomy and Plant Breeding, University of Tehran, Karaj, Iran; ^7^ Department of Production Animal Health, Faculty of Veterinary Medicine, University of Calgary, Calgary, AB, Canada

**Keywords:** bovine respiratory disease, RNA-seq, weighted gene co-expression network, protein-protein interaction, hub-hub genes

## Abstract

**Background:** Bovine respiratory disease (BRD) is the most common disease in the beef and dairy cattle industry. BRD is a multifactorial disease resulting from the interaction between environmental stressors and infectious agents. However, the molecular mechanisms underlying BRD are not fully understood yet. Therefore, this study aimed to use a systems biology approach to systematically evaluate this disorder to better understand the molecular mechanisms responsible for BRD.

**Methods:** Previously published RNA-seq data from whole blood of 18 healthy and 25 BRD samples were downloaded from the Gene Expression Omnibus (GEO) and then analyzed. Next, two distinct methods of weighted gene coexpression network analysis (WGCNA), i.e., module–trait relationships (MTRs) and module preservation (MP) analysis were used to identify significant highly correlated modules with clinical traits of BRD and non-preserved modules between healthy and BRD samples, respectively. After identifying respective modules by the two mentioned methods of WGCNA, functional enrichment analysis was performed to extract the modules that are biologically related to BRD. Gene coexpression networks based on the hub genes from the candidate modules were then integrated with protein–protein interaction (PPI) networks to identify hub–hub genes and potential transcription factors (TFs).

**Results:** Four significant highly correlated modules with clinical traits of BRD as well as 29 non-preserved modules were identified by MTRs and MP methods, respectively. Among them, two significant highly correlated modules (identified by MTRs) and six nonpreserved modules (identified by MP) were biologically associated with immune response, pulmonary inflammation, and pathogenesis of BRD. After aggregation of gene coexpression networks based on the hub genes with PPI networks, a total of 307 hub–hub genes were identified in the eight candidate modules. Interestingly, most of these hub–hub genes were reported to play an important role in the immune response and BRD pathogenesis. Among the eight candidate modules, the turquoise (identified by MTRs) and purple (identified by MP) modules were highly biologically enriched in BRD. Moreover, *STAT1*, *STAT2*, *STAT3*, *IRF7*, and *IRF9* TFs were suggested to play an important role in the immune system during BRD by regulating the coexpressed genes of these modules. Additionally, a gene set containing several hub–hub genes was identified in the eight candidate modules, such as *TLR2*, *TLR4*, *IL10*, *SOCS3*, *GZMB*, *ANXA1*, *ANXA5*, *PTEN*, *SGK1*, *IFI6*, *ISG15*, *MX1*, *MX2*, *OAS2*, *IFIH1*, *DDX58*, *DHX58*, *RSAD2*, *IFI44*, *IFI44L*, *EIF2AK2*, *ISG20*, *IFIT5*, *IFITM3*, *OAS1Y*, *HERC5*, and *PRF1*, which are potentially critical during infection with agents of bovine respiratory disease complex (BRDC).

**Conclusion:** This study not only helps us to better understand the molecular mechanisms responsible for BRD but also suggested eight candidate modules along with several promising hub–hub genes as diagnosis biomarkers and therapeutic targets for BRD.

## Introduction

Bovine respiratory disease (BRD) is the most common and costly infectious disease in the beef and dairy cattle industry. It causes 70–80% morbidity and 40–50% mortality in feedlot cattle in the United States (Edwards, 2010; Tizioto et al., 2015). BRD is a multifactorial disease, and its onset is usually associated with stress factors (nutritional or environmental risk factors) and the presence of infectious agents (Gagea et al., 2006; Grissett et al., 2015). Stress factors such as weaning, shipping distance, and commingling that negatively affect the immune system, can predispose cattle to a primary infection (Snowder et al., 2006; Timsit et al., 2016b). Infection is commonly caused by bovine respiratory disease complex (BRDC) including the viral and bacterial pathogens, which can affect the upper and lower respiratory system (Ellis, 2001; Caswell, 2014; Kirchhoff et al., 2014). Clinical diagnosis of BRD is made by visual observations and is usually based on clinical signs such as high rectal temperature, depression/lethargy, nasal or ocular discharge, increased respiration rate, reduced feed intake, and reduced average daily gain (Amrine et al., 2013; Behura et al., 2017). However, this method has low detection sensitivity and specificity, and the diagnosis is often made without identifying the cause of the disease (White and Renter, 2009; Timsit et al., 2016a). On the other hand, among the animals that are vaccinated against BRD, approximately 75% of them are protected (Hodgins et al., 2002), and the animals that are diagnosed based on clinical signs are treated with antimicrobials (Wilkinson, 2009). Moreover, excessive use of antimicrobial therapies for BRD facilitates the antibiotic resistance of microbes (Schaefer et al., 2007; Portis et al., 2012). In addition to vaccination and antimicrobials, other intervention methods such as nutritional manipulation and processing procedures have a limited effect on reducing morbidity and mortality rates, and despite extensive studies, BRD is still an issue (Taylor et al., 2010). Although the predisposing factors, viral and bacterial agents that cause BRD are relatively well known, the pathogenic mechanisms of BRD, the molecular immune response of the host to infection, and their association with the disease outcomes are not fully understood yet (Taylor et al., 2010; Johnston et al., 2019). Also, due to insufficient knowledge of the disease mechanisms, it is not possible to develop an effective method to identify animals with BRD (Sun et al., 2020). Therefore, understanding the infection dynamics and identification of new candidate biomarkers involved in BRD can help to better understanding the molecular mechanisms of BRD.

Functional genomic methods such as RNA-sequencing-based transcriptomics can provide a global gene expression profile, and their use in BRD studies can accelerate the understanding of disease mechanisms and the development of diagnosis (Rai et al., 2015). Several transcriptome studies have been performed on BRD in various tissues, such as the lung, bronchial lymph nodes (Behura et al., 2017; Johnston et al., 2019), and whole blood (Jiminez et al., 2021; Scott et al., 2021). For example, Sun et al. (2020) reported differentially expressed genes (DEGs) in the blood tissue as biomarkers to recognize BRD cattle at entry, such as *MX1*, *IFIT3*, *ISG15*, *OAS2*, and *IFI6* involved in the interferon signaling pathway. Moreover, Tizioto et al. (2015) identified 142 DEGs that were located in quantitative trait locus regions associated with BRD risk. The most common application of differential expression analysis is to identify DEGs between different conditions. On the other hand, it is known that the differential expression analysis focuses only on the individual effect of genes. However, genes and gene products do not work individually but interact in complex gene networks (Liu et al., 2020). Therefore, individual evaluation of gene expression may not explain the cause of complex diseases such as BRD.

Systems biology is one of the suitable methods to better understand the mechanism of diseases (Darzi et al., 2021) and other complex traits (Salleh et al., 2018). In systems biology, there are various computational methods based on the network approach, and one of the fundamental aspects of the network approach in systems biology is the construction of gene coexpression networks using high-throughput gene expression data (Kadarmideen and Watson-Haigh, 2012). In this regard, one of the most widely used methods for building gene coexpression networks is weighted gene coexpression network analysis (WGCNA) (Langfelder and Horvath, 2008). The WGCNA method identifies clusters of coexpressed genes (also called a module) based on correlation patterns between expression profiles of genes across samples (Langfelder and Horvath, 2008). Furthermore, the WGCNA identifies highly connected genes (hub genes) by calculating intramodular gene connectivity, which are centrally in their modules and can be involved in important roles during the disease (Bakhtiarizadeh et al., 2018). The WGCNA approach has been used successfully in different disease studies in humans (Wang Y. et al., 2020), cattle (Yan et al., 2020), swine (Wilkinson et al., 2016), mice (Rangaraju et al., 2018), chickens (Liu and Cai, 2017), and sheep (Kadarmideen et al., 2011). One of the most widely used methods of WGCNA is module–trait relationship analysis. In this method, after identifying the modules across samples, module–trait relationships are calculated according to the correlation between module eigengenes and traits of interest, and finally, significant modules are identified (Kommadath et al., 2014; Sabino et al., 2018). Moreover, WGCNA provides another unique network-based method called module preservation analysis. This method focuses on determining network topology changes across different conditions. For example, it can be checked whether the network density and topological pattern of the modules identified in normal samples (as a reference) are preserved in the disease samples (as a test) (Langfelder et al., 2011). In this regard, the differences in the topology of these two networks indicate a significant perturbation of the coexpression patterns by the disease. Thus, the nonpreserved modules between these conditions, as well as their hub genes may exert crucial roles in the pathological processes of the disease (Mukund and Subramaniam, 2015; Riquelme Medina and Lubovac-Pilav, 2016; Bakhtiarizadeh et al., 2020).

In the present study, we used previously published RNA-seq data (Jiminez et al., 2021) and the WGCNA method for constructing weighted gene coexpression networks to better understand the molecular regulatory mechanisms responsible for the immune response to BRD. It should be noted that in the current study we used two distinct WGCNA methods to identify key modules, their hub genes, hub–hub genes, and regulatory factors involved in BRD: 1) module–trait relationships analysis across all samples to identify significant highly correlated modules with clinical traits of BRD, and 2) module preservation analysis between healthy samples (as reference set) and BRD samples (as test set) to identify nonpreserved modules between these conditions. The main hypothesis was that significant highly correlated modules with clinical traits of BRD (identified by the first method) and nonpreserved modules between healthy and BRD samples (identified by the second method) may contain potential functionally related genes and identifies biological regulatory systems involved in pathological processes of BRD, and it is also expected that these two methods confirm each other’s results.

## Materials and Methods

### Datasets

RNA sequencing data from feedlot cattle with and without BRD were obtained from the Gene Expression Omnibus (GEO) database at the National Center for Biotechnology Information (NCBI) under the accession number of GSE162156. Moreover, clinical traits of BRD were obtained from the supplementary material section of the original paper (Jiminez et al., 2021) and then filtered for useful measurements. The data included samples from the whole blood of 25 and 18 mixed-breed beef heifers with and without BRD, respectively. An Illumina HiSeq 4000 platform was used to generate 43 paired-end (2 × 100 bp) libraries that included 18 healthy and 25 BRD samples. More information about the data can be found in the original paper (Jiminez et al., 2021).

### RNA-Seq Data Analysis and Preprocessing

Quality control of the raw sequencing data was performed using FastQC^1^ (version 0.11.9). Raw reads were then trimmed by removing low-quality bases and adaptor sequences using the Trimmomatic software (version 0.39) (Bolger et al., 2014) with the following options: ILLUMINACLIP: Adapter. fa:2:30:10, LEADING:20, TRAILING:20, and MINLEN:60. To confirm quality improvements, the clean reads were checked again using FastQC. Then the Hisat2 (version 2.2.1) (Kim et al., 2015) software was used for aligning the clean reads to the latest bovine reference genome (ARS-UCD1.2) with default parameters. To calculate counts of uniquely mapped reads to annotated genes based on the bovine GTF file (release 104), the python script HTSeq-count (version 0.13.5) (Anders et al., 2014) was applied using intersection-strict mode. All count files were then merged and finally, a raw gene expression matrix was created containing read counts information of all genes for all samples.

### Weighted Gene Coexpression Network Analysis

Raw gene expression matrix obtained from the previous steps was normalized to log-counts per million (log-cpm) using the “voom” function of the limma package (version 3.46.0) (Smyth, 2005). This normalization method opens access for the gene expression data generated by the RNA-seq analysis, to various computational methods, such as WGCNA (Law et al., 2014). Because the low-expressed or low-variance genes usually represent sampling noise and correlations based on these genes are not significant, genes with <1 CPM (counts per million) in at least five samples were removed. In addition, genes with a standard deviation >0.25 were selected for further analysis. Weighted gene coexpression network analysis was performed based on the functions of the WGCNA R package (version 1.70) (Langfelder and Horvath, 2008).

#### Module–Trait Relationships Analysis

To identify significant highly correlated modules with clinical traits of BRD, all 43 samples (18 healthy and 25 BRD) were used for module–trait relationships (MTRs) analysis. Because the gene coexpression analysis is very sensitive to outliers, the distance-based adjacency metrics of samples was calculated and samples with a standardized connectivity < −2.5 were removed, considered as an outlier. In addition, samples and genes with >50% missing entries and genes with zero variance were identified and excluded from the WGCNA analysis. In this study, a signed weighted coexpression network was constructed in which correlation values between 0 and 1 are considered and values <0.50 are considered as negative correlation, and values >0.50 are considered as positive correlation (van Dam et al., 2017). Signed networks considers only positively correlated genes, and especially, network construction based on this method leads to more significantly enriched modules (Mason et al., 2009). Furthermore, bi-weight mid-correlation coefficient was used for the coexpression network construction since it is more robust to outliers in comparison to the Pearson correlation (Zheng et al., 2014). Briefly, a correlation matrix of expression values was constructed using pairwise bi-weight mid-correlation coefficients between all pairs of genes across the selected samples. Then, the correlation matrix at *β* = 10 as a soft threshold (power) was transformed into weighted adjacency matrix. Subsequently, the weighted adjacency matrix was transformed into topological overlap matrix (TOM), which considers each pairs of genes concerning all other genes by comparing their connections with all other genes in the network (interconnectedness). In other words, the genes in a module share strong interconnectedness (Zhang and Horvath, 2005; Li and Horvath, 2006; Yip and Horvath, 2007). Finally, average linkage hierarchical clustering analysis was performed by the topological overlap-based dissimilarity matrix (1-TOM) as input, and modules were identified by dynamic hybrid tree cutting algorithm. Then the modules with the highly correlated eigengenes were merged. The above steps were performed using automatic, one-step network construction and module detection function “blockwiseModules” of the WGCNA R package with the following parameters: power = 10, corType = “bicor,” maxBlockSize = 12,000, networkType = “signed,” TOMType = “signed,” minModuleSize = 30, reassignThreshold = 0, and mergeCutHeight = 0.25. Next, in order to identify the BRD-related modules, the correlation between the clinical traits of BRD and module eigengenes (the first principal component of the expression matrix for a given module) was taken using Pearson correlation coefficient. The cutoff of significant moderately or highly correlated modules with clinical traits of BRD was defined as *p*-value < 0.05 and 0.30 < |*R*| < 0.50, and *p-*value < 0.05 and |*R*| > 0.50, respectively. Moreover, gene significance (GS), which is a criterion for biological association of a gene with an interest trait was calculated for each gene through the correlation between gene expression profile and clinical traits of BRD.

#### Module Preservation Analysis

In this method, based on the assumption that BRD may cause a topological change in the coexpression patterns of the healthy samples, and that nonpreserved modules between the healthy and disease samples may be biologically related to BRD, the healthy samples (*n* = 18) were selected as a reference set for construction the coexpression network and modules detection. So, after outlier detection, removing them, and set *β* = 13 as a soft threshold, automatic module detection function “blockwiseModules” of the WGCNA was used for a signed network construction, as well as identification of modules in healthy samples with following parameters: networkType = “signed,” TOMType = “signed,” corType = “bicor,” mergeCutHeight = 0.25, power = 13, maxBlockSize = 12,000, minModuleSize = 30, and reassignThreshold = 0. After identifying the modules, module preservation analysis was performed using the “module Preservation” function of WGCNA R package to investigate whether the network density and connectivity patterns of the modules were preserved between the healthy and BRD samples. For this purpose, two composite preservation statistics were investigated using a permutation test (based on 200 random permutations). The first preservation composite statistic was Zsummary that was calculated from a combination of several preservation statistics, which investigated whether the mean connection strength among all genes in a module (known as network density) identified in the healthy samples remain highly connected in the disease samples and it also evaluates whether the sum of the connection strengths for a gene with other network genes (known as connectivity) in the healthy samples are similar in the disease samples (Langfelder et al., 2011). A higher value of Zsummary indicates strong preservation between conditions (healthy vs. BRD). However, Zsummary increases with increasing module size, Therefore, it is strongly dependent on the module size (Langfelder et al., 2011). The second preservation composite statistic used is medianRank, which is a module size-independent statistic; this rank-based measure relies on observed preservation statistics. Unlike Zsummary, modules with low medianRank values are highly preserved between conditions. In this study, modules with Zsummary > 10 and medianRank < 8, 5 < Zsummary ≤ 10 and medianRank < 8, and Zsummary ≤ 5 or medianRank ≥ 8 were considered as highly-preserved, semipreserved, and nonpreserved, respectively.

### Functional Enrichment Analysis and Transcription Factors Prediction

To determine which modules are biologically related to BRD, all genes in each module were analyzed for Gene Ontology (GO) and Kyoto Encyclopedia of Genes and Genomes (KEGG) pathways using the Enrichr web tool (Chen et al., 2013). The cutoffs of significant terms were defined as adjusted *p-*value <0.05 (correction by the Benjamini–Hochberg method). Moreover, to identify potential regulatory factors in the modules, the genes of each module were aligned to bovine transcription factors (TFs) set from the AnimalTFDB3.0 database (Hu et al., 2018).

### Hub Genes Identification and Protein–Protein Interaction Network Construction

Highly connected genes (hub genes) in a coexpression module are suitable candidates for explaining behavior and biological function of that module. In other words, highly connected intramodular hub genes have the highest degree of connection in a module and are central to modules in a network, and compared with other genes, they have more biological relevance to the module functions (van Dam et al., 2017). In this regard, module membership (MM) also known as eigengene-based connectivity *k*_*ME*_ for each gene was calculated by the WGCNA R package through the correlation between the gene expression profile and the module eigengenes. Next, this criterion was used to identify hub genes in the significant highly correlated modules with clinical traits of BRD that were identified by the MTRs method and nonpreserved modules that were identified by the MP method. In fact, the MM assesses how well the genes of a module correlate with the characteristics of that module. Genes with *k*_*ME*_ ≥ 0.7 were considered as highly connected hub genes in the respective modules. Furthermore, to investigate the connections of proteins encoded by the hub genes, Search Tool for the Retrieval of Interacting Genes (STRING) database (version 11.0) (Szklarczyk et al., 2018) was used and the protein–protein interaction (PPI) network of the hub genes was attained for further analysis.

### Hub–Hub Gene Detection and Network Visualization

For detection of the highly connected and central genes in the PPI network based on the hub genes (hub–hub genes), cytoHubba application (version 0.1) was used (Chin et al., 2014). This application is a cytoscape plugin and explores important genes and subnetworks in a given biological network such as the PPI network by several topological analysis methods including local-based and global-based methods. Local-based methods only considers the direct neighborhood of a gene, including degree (Deg), maximum neighborhood component (MNC), density of maximum neighborhood component (DMNC), clustering coefficient (CC), and maximal clique centrality (MCC) methods. Global-based methods focus on the shortest paths, such as closeness (Clo), eccentricity (EcC), radiality (Rad), bottleneck (BN), stress (Str), and betweenness (BC) methods (Chin et al., 2014). These 12 topological analysis methods were used separately to rank 60 important genes in each PPI network that were derived from the hub genes. Next, for rank aggregation of important genes lists, RankAggreg R package (version 0.6.6) was used based on cross-entropy (CE) algorithm and genetic algorithm (GA) (Pihur et al., 2009). Finally, the common genes between these two methods were considered as hub–hub genes. Significant highly correlated modules (identified by MTRs) and nonpreserved modules (identified by MP) that were biologically associated with BRD were visualized using Cytoscape (version 3.7.1) (Cline et al., 2007).

## Results

### RNA-Seq Data Analysis

**FIGURE 1:**
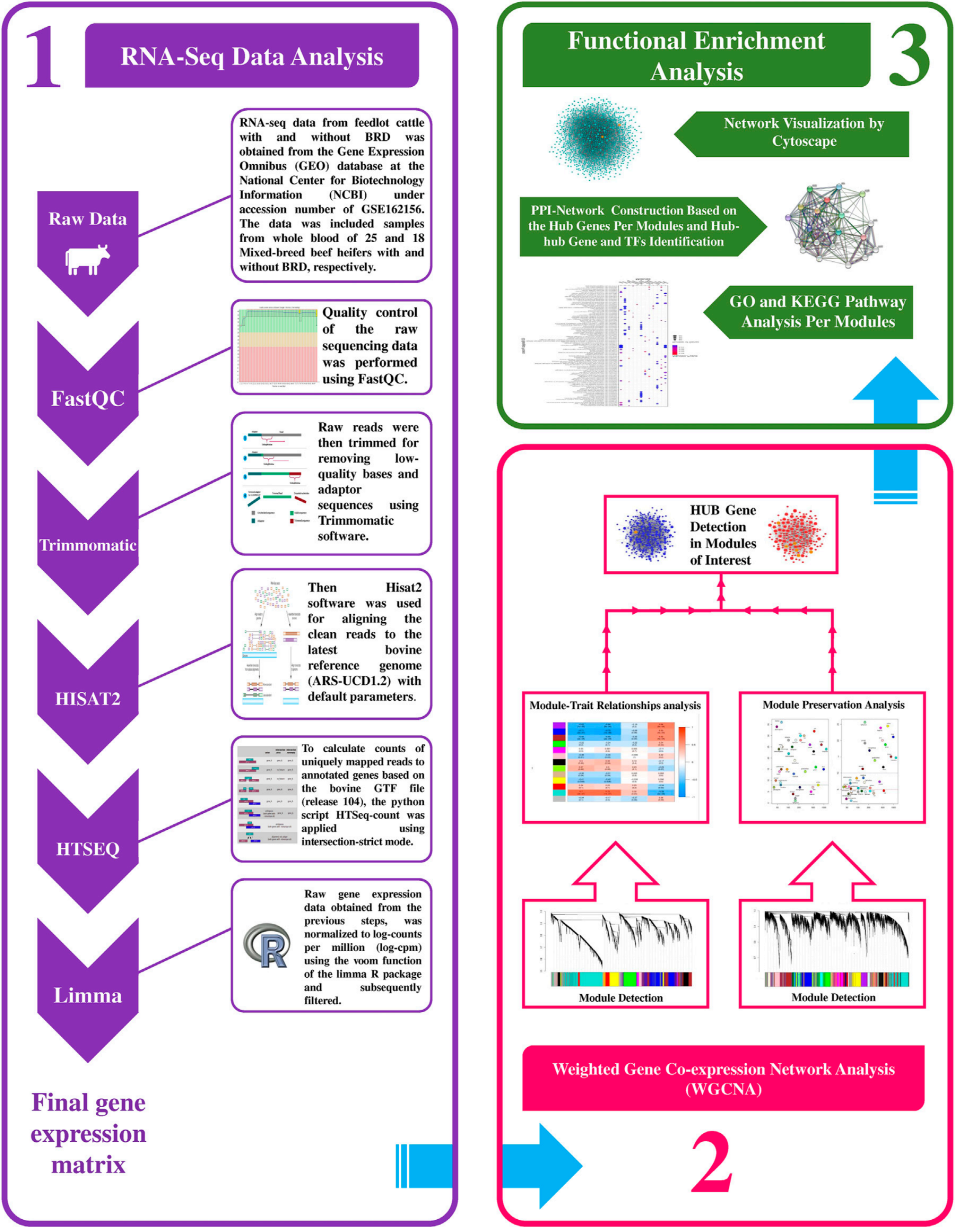
Schematic pipeline for RNA-seq data analysis and weighted gene coexpression network construction in this study.

A summary for the RNA-seq data analysis pipeline and the steps for constructing the weighted gene coexpression network is presented in Figure 1. RNA-seq data included 43 samples (18 healthy and 25 BRD), and there was a mean of 31.781 million paired-end (2 × 100 bp) reads per sample. A total of 1.366 billion reads were analyzed and after trimming, a total of 1.352 billion clean reads were obtained (approximately 31.469 million clean reads per sample). On average, 95% of all clean reads were mapped to the bovine reference genome (ranging from 92 to 97%). Moreover, a mean of 82% of all clean reads were uniquely mapped to the bovine reference genome. The details about RNA-seq data, trimming, and mapping summary of all samples is provided in Supplementary Table S1. Finally, after applying various parameters to filter the low-expressed and low-variance genes, a total of 10,099 genes were further used in the WGCNA analysis. A list of filtered genes along with the normalized values of their expression is provided in Supplementary Table S2.

### Module–Trait Relationship Analysis

**FIGURE 2:**
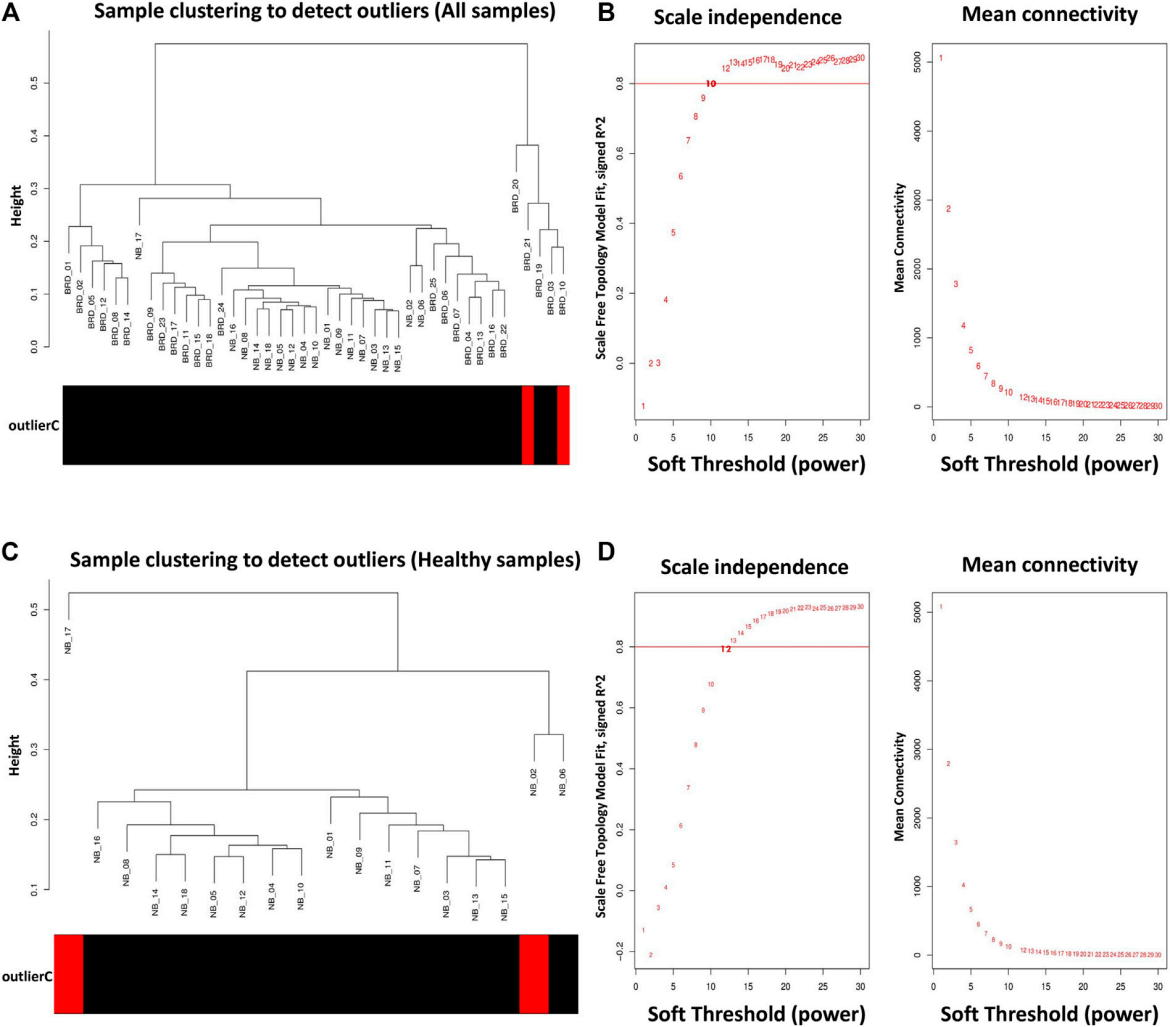
Sample clustering to detect outliers and network topology analysis. **(A)** All samples except GSM4943645 and GSM4943656 were clustered and then selected for module–trait relationships analysis. **(B)** Scale-free topology fitting index (left) and mean connectivity (right) for different soft-threshold powers (*β*). For module–trait relationships analysis, coexpression network was constructed at *β* = 10 whose scale-free topology fitting index (*R* ^*2*^) was ≥0.80. **(C)** All samples except GSM4943661 and GSM4943667 were clustered and then selected for module preservation analysis. **(D)** Scale-free topology fitting index (left) and mean connectivity (right) for different soft-threshold powers (*β*). For module preservation analysis, co-expression network was constructed at *β* = 13 whose scale-free topology fitting index (*R* ^*2*^) was >0.80.

**FIGURE 3:**
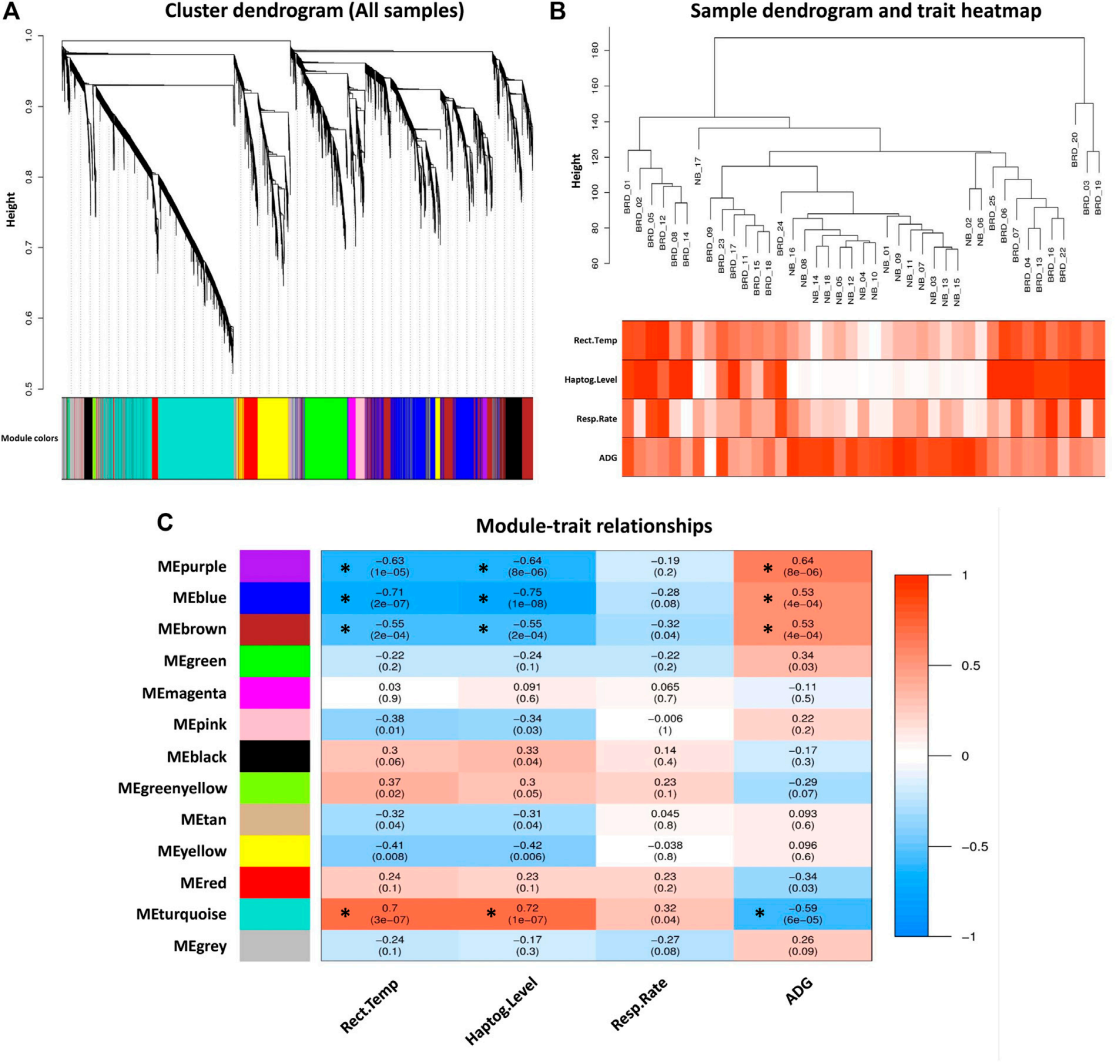
Module–trait relationships analysis. **(A)** Gene hierarchical clustering dendrogram of 12 detected modules based on a dissimilarity (1-TOM) measure across all samples, the y-axis represents the coexpression distance and the x-axis represents the genes. The branches indicate the modules, and each module is marked with a separate color, the gray module encompass genes that are not assigned to any of the modules. **(B)** Sample dendrogram and trait heatmap of clinical traits related to BRD across all samples. The gradient from white to red indicates the low to high level of the respective traits in the samples. **(C)** Module–trait relationships between detected modules and clinical traits of BRD. Module–trait relationships are obtained by calculating the correlation between the traits and the module eigengenes. The red and blue colors indicate strong positive correlation and strong negative correlation, respectively. Rows represent module eigengene (ME) and columns indicate clinical traits of BRD. Rect. Temp, rectal temperature (°C); Haptog. Level, haptoglobin level (g/L); Resp. Rate, respiratory rate (per min), ADG, average daily gain. Asterisks corresponds to significant highly correlated values.

To prevent the negative effects of outlier samples on gene coexpression network analysis, after identifying the outliers, two samples (GSM4943645 and GSM4943656) with a standardized connectivity score < −2.5 were removed (Figure 2A). The weighted adjacency matrix was constructed at *β* = 10 whose scale-free topology fitting index (*R* ^*2*^) was ≥0.80 (Figure 2B). After network construction, 12 coexpression modules (excluding grey module with 690 uncorrelated genes) were identified through hierarchical clustering and dynamic hybrid tree cutting with an average size of 784 genes. The turquoise and tan modules were the largest and smallest module with 2,592 and 72 genes each, respectively (Supplementary Table S3). In Figure 3A, a clustering dendrogram is presented in which the branches represent the modules that are labeled with a specific color by the WGCNA R package. Clinical traits related to BRD that were used in MTRs included clinical signs measurements of BRD such as rectal temperature (°C), haptoglobin level (g/L), respiratory rate (per min), and average daily gain. The sample dendrogram and trait heatmap of clinical traits related to BRD across all samples are presented in Figure 3B. The results of MTRs indicate that the rectal temperature, haptoglobin level, average daily gain, and respiratory rate have eight, eight, six, and two significant modules, respectively. Among the significant modules are as follows: MEpurple (*R* = −0.63, *p* = 1e−05), MEblue (*R* = −0.71, *p* = 2e−07), MEbrown (*R* = −0.55, *p* = 2e−04), and MEturquoise (*R* = 0.7, *p* = 3e−07) modules were significantly highly correlated and MEyellow (*R* = −0.41, *p* = 0.008), MEtan (*R* = −0.32, *p* = 0.04), MEgreenyellow (*R* = 0.37, *p* = 0.02), and MEpink (*R* = −0.38, *p* = 0.01) modules were significantly moderately correlated with rectal temperature, respectively (Figure 3C). Also, MEpurple (*R* = −0.64, *p* = 8e−06), MEblue (*R* = −0.75, *p* = 1e−08), MEbrown (*R* = −0.55, *p* = 2e−04), and MEturquoise (*R* = 0.72, *p* = 1e−07) modules were significantly highly-correlated and MEyellow (*R* = −0.42, *p* = 0.006), MEtan (*R* = −0.31, *p* = 0.04), MEblack (*R* = 0.33, *p* = 0.04), and MEpink (*R* = −0.34, *p* = 0.03) modules were significantly moderately correlated with haptoglobin level, respectively (Figure 3C). Moreover, MEpurple (*R* = 0.64, *p* = 8e−06), MEblue (*R* = 0.53, *p* = 4e−04), MEbrown (*R* = 0.53, *p* = 4e−04), and MEturquoise (*R* = −0.59, *p* = 6e−05) modules were significantly highly correlated, and MEred (*R* = −0.34, *p* = 0.03) and MEgreen (*R* = 0.34, *p* = 0.03) modules were significantly moderately correlated with average daily gain, respectively (Figure 3C). The MEturquoise (*R* = 0.32, *p* = 0.04) and MEbrown (*R* = −0.32, *p* = 0.04) modules were significantly moderately correlated with respiratory rate, respectively (Figure 3C), but no significant highly correlated modules were found for this trait. Then, the significant highly correlated modules were selected for downstream analysis. Briefly, the turquoise, blue, brown, and purple modules with module sizes of 2,592, 1,691, 1,214, and 141 genes, respectively, were identified as significantly highly correlated modules with rectal temperature, haptoglobin level, and average daily gain (Figure 3C).

### Functional Enrichment Analysis of Highly Correlated Modules

**FIGURE 4:**
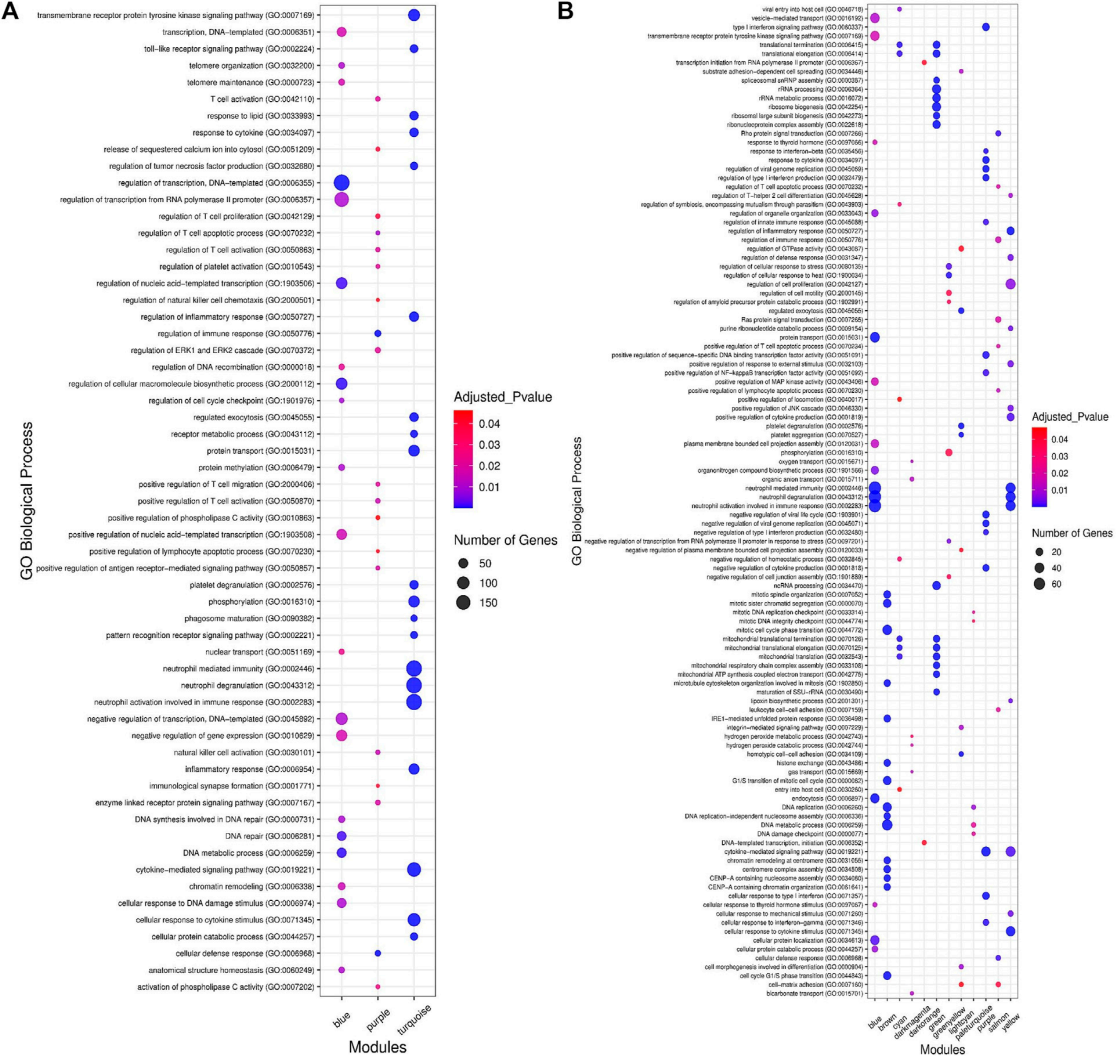
Functional enrichment analysis results. **(A)** The top 20 significant biological processes for significant highly correlated and biologically related modules to bovine respiratory disease (BRD). Color and size and each point represent −log2(FDR) and number of genes for each term, respectively. **(B)** The top 15 significant biological processes for significant enriched nonpreserved modules. Color and size and each point represent −log2(FDR) and number of genes for each term, respectively.

In order to understand the biological performance of the significant highly correlated modules with clinical traits of BRD, functional enrichment analysis was performed and a total of 356 biological process and 129 KEGG pathways were significantly enriched in the respective modules. The turquoise module had the highest number of enriched terms and pathways, including 305 biological processes and 116 KEGG pathways. The most significant GO term and KEGG pathway in the turquoise module were “neutrophil-mediated immunity” (GO:0002446, adjusted *p*-value = 7.45E−55) and “Lysosome” (adjusted *p*-value = 5.16E−13), respectively. On the other hand, 19 biological processes and 13 KEGG pathways were significantly enriched in the purple module. The most significant GO term and KEGG pathway in the purple module were “cellular defense response” (GO:0006968, adjusted *p-*value = 2.27E−09) and “natural killer cell-mediated cytotoxicity” (adjusted *p*-value = 2.09E−06), respectively. Only 32 biological processes were enriched in the blue module, and no biological process or KEGG pathway was significantly enriched in the brown module. The top 20 significant biological process terms for turquoise, blue, and purple modules are presented in Figure 4A. Moreover, the complete information of the functional enrichment analysis for the significant highly correlated modules with clinical traits of BRD is provided in Supplementary Table S4. Based on the functional enrichment analysis, among the significant highly correlated modules with clinical traits of BRD, turquoise and purple modules were associated with BRD mechanisms and host immune response. To identify potential TFs that may control transcription of coexpressed genes in the modules, a total number of 100 and 11 TFs were found in the turquoise and purple modules, respectively (Supplementary Table S5).

### Hub and Hub–Hub Gene Detection in Highly Correlated Modules

**FIGURE 5:**
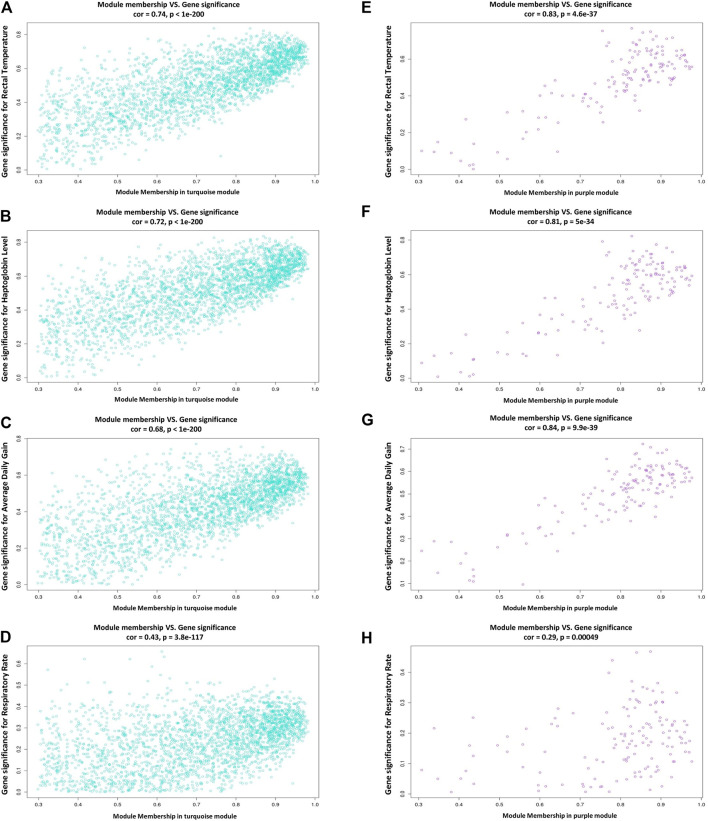
Scatterplots of module membership (MM) versus gene significance (GS) plots. **(A–D)** module membership versus gene significance for rectal temperature, haptoglobin level, average daily gain, and respiratory rate in the turquoise module, respectively. **(E–H)** module membership versus gene significance for rectal temperature, haptoglobin level, average daily gain, and respiratory rate in the purple module, respectively.

**FIGURE 6:**
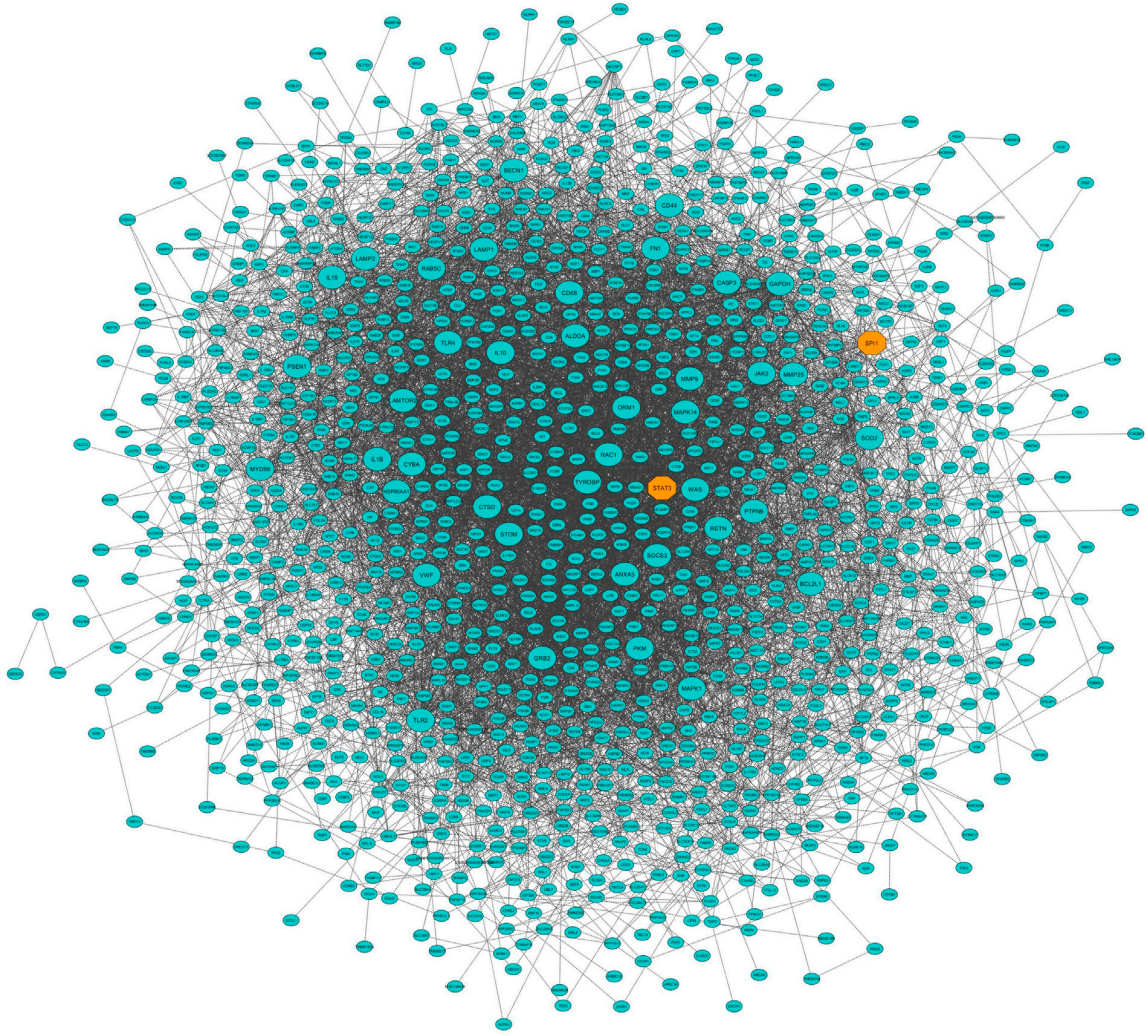
Protein–protein interaction (PPI) network based on the hub genes of the turquoise module (identified by module–trait relationships method). Larger nodes and orange octagons represent hub–hub genes and transcription factors, respectively.

In this study, coexpressed genes in both turquoise and purple modules (as significant highly correlated as well as biologically related modules with BRD) were further evaluated. The MM versus GS plots of these modules are presented in Figure 5, which shows a strong correlation between GS and MM. In other words, the most significant genes with clinical traits of BRD are often the central genes in the respective modules. More information about GS for clinical traits of BRD can be found in Supplementary Table S6. Highly connected intramodular hub genes often have high levels of MM and may play an important role in BRD. So, in this regard, a total of 1,476 and 114 hub genes were identified in turquoise and purple modules, respectively (Supplementary Table S7). Then, the connection of proteins encoded by these hub genes in each module was examined and the PPI network related to the hub genes in turquoise and purple modules was obtained. Interestingly, PPI networks based on the hub genes in the turquoise and purple modules were of high density based on the STRING database information. The PPI network based on the hub genes in the turquoise module is presented in Figure 6. Hub–hub genes, which were highly connected in the respective coexpression modules and also are central genes in the hub genes-based PPI networks, can be considered as prognostic and therapeutic targets in BRD development. Here, a total of 42 and 23 hub–hub genes were found in turquoise and purple modules, respectively (Table 1).

**TABLE 1:** List of the hub–hub genes in the turquoise and purple modules as significant highly correlated and biologically related modules with bovine respiratory disease (BRD) along with their module memberships (MM) and gene significance (GS) for rectal temperature (identified by module–trait relationships analysis).

Module					
Turquoise			Purple		
Genes	MM	GS	Genes	MM	GS
GAPDH	0.85	0.56	PRF1	0.97	−0.54
IL10	0.93	0.68	KLRK1	0.92	−0.71
STAT3	0.89	0.65	IL2RB	0.95	−0.62
MAPK1	0.81	0.66	LCK	0.85	−0.55
CASP3	0.81	0.51	ITK	0.82	−0.60
LAMP1	0.86	0.56	EOMES	0.82	−0.64
FN1	0.72	0.45	KLRD1	0.88	−0.52
MAPK14	0.91	0.64	CD40LG	0.81	−0.52
TLR4	0.93	0.67	NCR1	0.91	−0.58
TLR2	0.89	0.50	CCL5	0.92	−0.49
CD68	0.90	0.65	LOC618565	0.74	−0.36
RAC1	0.76	0.37	TBX21	0.93	−0.64
CD44	0.77	0.49	CD8A	0.88	−0.62
JAK2	0.87	0.55	RUNX3	0.83	−0.68
IL1B	0.86	0.50	XCL2	0.74	−0.30
CTSD	0.85	0.65	CCR8	0.95	−0.64
MMP9	0.93	0.62	CX3CR1	0.90	−0.57
GRB2	0.82	0.42	SH2D1A	0.86	−0.65
ANXA5	0.81	0.46	GPR55	0.82	−0.76
TYROBP	0.96	0.66	CTSW	0.92	−0.57
PTPN6	0.79	0.51	KIR2DS1	0.72	−0.34
RAB5C	0.90	0.57	NKG7	0.93	−0.54
SOD2	0.93	0.77	CD96	0.95	−0.61
BECN1	0.81	0.41	—	—	—
WAS	0.94	0.68	—	—	—
LAMTOR2	0.87	0.75	—	—	—
CYBA	0.86	0.56	—	—	—
SOCS3	0.94	0.66	—	—	—
ALDOA	0.93	0.67	—	—	—
SPI1	0.96	0.61	—	—	—
RETN	0.78	0.47	—	—	—
MYD88	0.93	0.68	—	—	—
VWF	0.77	0.63	—	—	—
PKM	0.97	0.66	—	—	—
ORM1	0.80	0.62	—	—	—
STOM	0.91	0.52	—	—	—
BCL2L1	0.81	0.70	—	—	—
HSP90AA1	0.89	0.72	—	—	—
MMP25	0.96	0.67	—	—	—
LAMP2	0.91	0.57	—	—	—
PSEN1	0.82	0.47	—	—	—
IL18	0.81	0.58	—	—	—

Note that the rectal temperature is one of the most important and widely used clinical signs of BRD.

### Module Preservation Analysis

**FIGURE 7:**
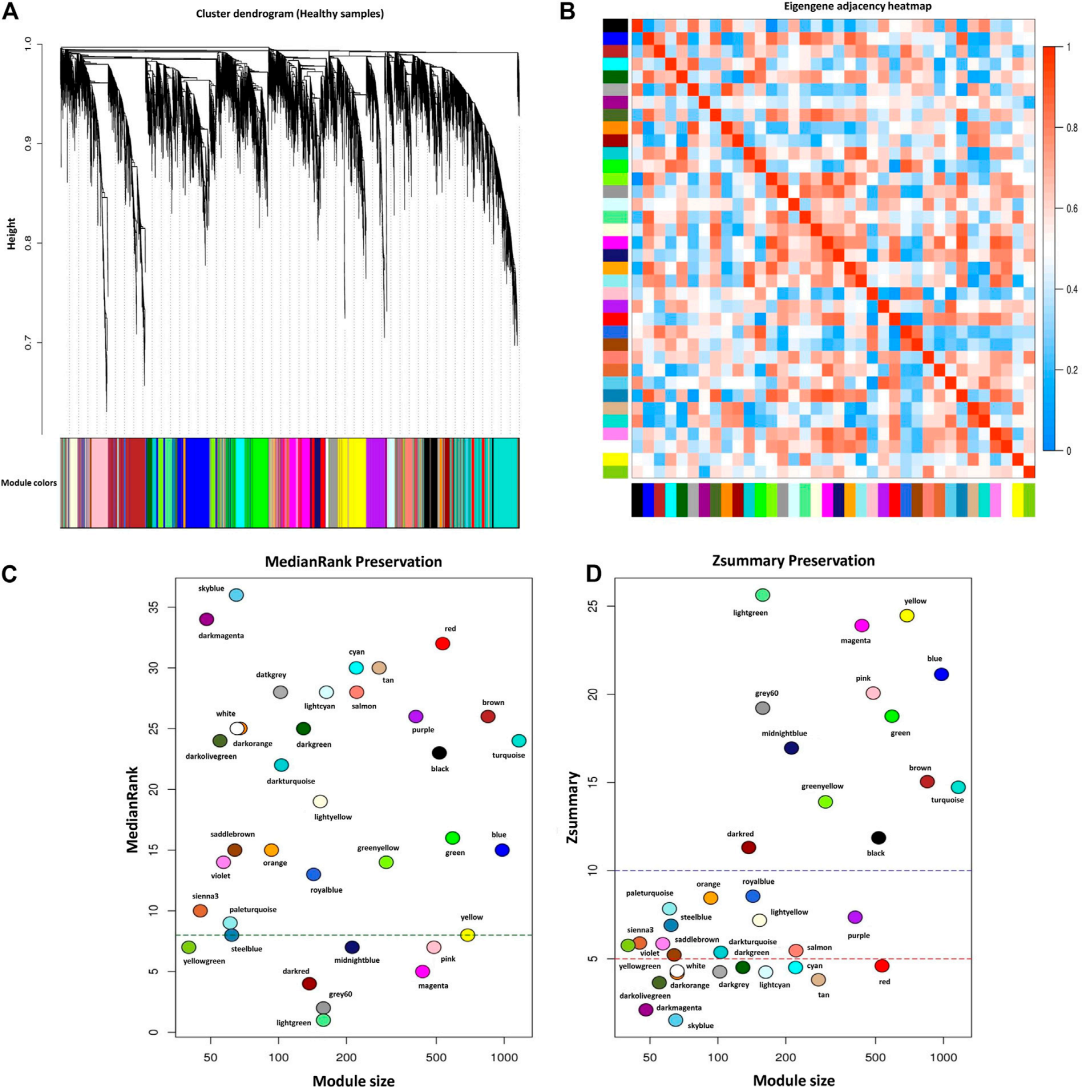
Module preservation analysis. **(A)** Gene hierarchical clustering dendrogram of 36 detected modules based on a dissimilarity (1-TOM) measure across healthy samples as reference set, the y-axis represents the coexpression distance and the x-axis represents the genes. The branches indicate the modules, and each module is marked with a separate color, the gray module encompass genes that are not assigned to any of the modules. **(B)** Eigengene adjacency heatmap indicate relationship among all the modules. **(C)** The medianRank preservation statistics of the modules. The y-axis and the x-axis represent medianRank values and module size, respectively. Each point indicates a module labeled by a respective color. The green dashed line represents the medianRank threshold (medianRank ≥8). **(D)** The Zsummary preservation statistics of the modules. The y-axis and the x-axis represent Zsummary values and module size, respectively. Each point indicates a module labeled by a respective color. The red dashed line represents the Zsummary threshold (Zsummary ≤5). Modules with Zsummary ≤5 or medianRank ≥8 were considered as nonpreserved between healthy and BRD conditions.

For module preservation analysis, healthy samples were used as a reference set to construct the weighted gene coexpression network. Two samples, GSM4943661 and GSM4943667 were identified as outliers based on the distance adjacency metrics of samples and then removed (Figure 2C). For network construction, the soft threshold power was set to 13 which showed high scale-free topology (*R* ^*2*^ > 0.80; Figure 2D). Using WGCNA, a total of 36 modules were identified in the healthy samples. The modules had different sizes ranging from 40 in the yellowgreen module to 1,163 in the turquoise module. In addition, the grey module contained 247 genes that were not assigned to any of the other modules (Supplementary Table S8). The identified modules in the healthy samples with different colors as branches of the hierarchical clustering dendrogram and the relationship between them are presented in Figures 7A,B, respectively. Next, for the module preservation analysis, we used the BRD samples as a test set to investigate whether the network density and connectivity pattern of the modules identified in the healthy samples were preserved in the BRD samples. The results of the preservation analysis showed that based on the thresholds set in the *Materials and methods* section, six modules, including lightgreen (Zsummary = 26, medianRank = 1), magenta (Zsummary = 24, medianRank = 5), pink (Zsummary = 20, medianRank = 7), grey60 (Zsummary = 19, medianRank = 2), midnightblue (Zsummary = 17, medianRank = 7), and darkred (Zsummary = 11, medianRank = 4), were identified as highly preserved modules, and one module, yellowgreen (Zsummary = 5.8, medianRank = 7) was identified as a semipreserved module (Figure 7C). Interestingly, in agreement with our hypothesis, of the 36 modules identified, 29 modules were non-preserved between healthy and BRD samples, indicating that their connectivity pattern and topological structure have been affected and changed by the BRD (Figure 7C). Skyblue (Zsummary = 1.5, medianRank = 36), darkmagenta (Zsummary = 2.1, medianRank = 34), and darkolivegreen (Zsummary = 3.6, medianRank = 24) modules were identified as the most non-preserved modules between healthy and BRD samples, respectively (Supplementary Table S9).

### Functional Enrichment Analysis of Highly Preserved, Semipreserved, and Nonpreserved Modules

To understand the biological significance and the functional difference between highly preserved, semipreserved, and nonpreserved modules, all the 36 modules were subjected to GO terms and KEGG pathway analysis, and a total of 521 biological processes and 158 KEGG pathways were significantly enriched. Enrichment analysis of four highly preserved modules that included magenta, pink, grey60, and midnightblue revealed a total of 108 and 27 biological processes and KEGG pathways, respectively. Among them, the magenta module with 37 biological processes and 17 KEGG pathways was identified as the most significant enriched module. The other two highly preserved modules, including lightgreen and darkred, showed no biological process or KEGG pathway enrichment. Moreover, one biological process was enriched in the yellowgreen module as the only semipreserved module. The complete information of the functional enrichment analysis for the highly preserved and semipreserved modules is provided in Supplementary Table S10. On the other hand, among the nonpreserved module, 13 modules including blue, brown, cyan, darkmagenta, darkorange, green, greenyellow, lightcyan, paleturquoise, purple, red, salmon, and yellow were enriched in at least one biological process or KEGG pathway (significantly enriched nonpreserved modules). Furthermore, functional enrichment analysis of the significantly enriched nonpreserved modules identified a total of 412 biological processes and 131 KEGG pathways. The top 15 significant biological process terms for significantly enriched nonpreserved modules are presented in Figure 4B. In addition, the complete information of the functional enrichment analysis for the nonpreserved modules is provided in Supplementary Table S11. Based on the enrichment results, among the significantly enriched nonpreserved modules, six nonpreserved modules including blue, greenyellow, purple, red, salmon, and yellow were associated with pathogenic mechanisms of BRD and immune response. Also, based on the bovine transcription factors set from the AnimalTFDB3.0 database, a total number of 37, 18, 25, 55, 26, and 39 TFs were identified in blue, greenyellow, purple, red, salmon, and yellow modules, respectively (Supplementary Table S12).

### Hub and Hub–Hub Gene Identification in Nonpreserved Modules

**FIGURE 8:**
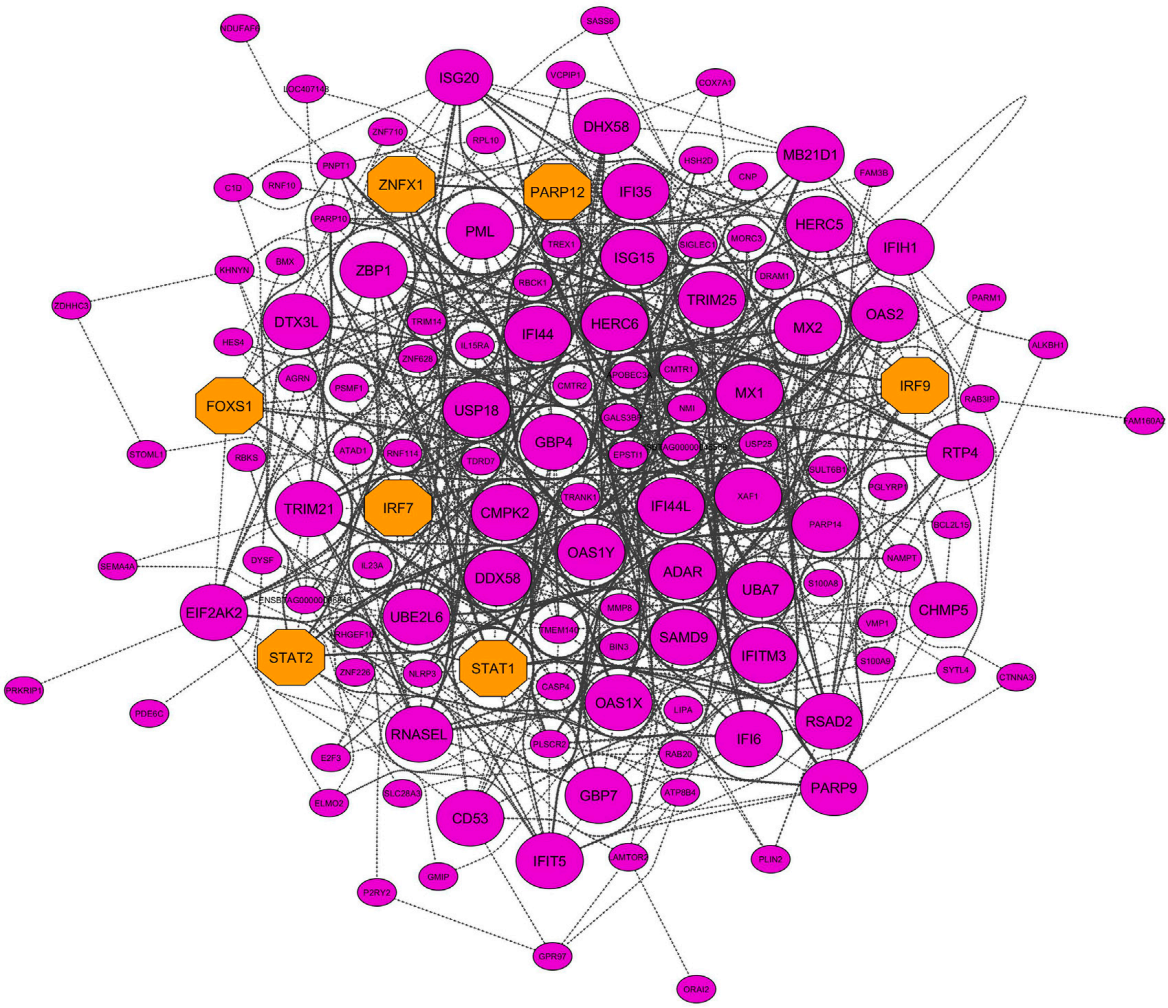
PPI network based on the hub genes of the purple module (identified by module preservation method). Larger nodes and orange octagons represent hub–hub genes and transcription factors, respectively.

Six nonpreserved modules were identified as potential candidate modules and biologically related to BRD based on the module preservation and functional enrichment analysis, respectively. Then, MM measures were used to identify intramodular hub genes as important and central genes in these modules. A total number of 326, 251, 216, 145, 131, and 99 hub genes were detected in blue, yellow, red, purple, greenyellow, and salmon modules, respectively. The complete list of the hub genes in the nonpreserved modules can be found in Supplementary Table S13. Hub genes-based PPI networks extracted from the STRING database identified 303 nodes (proteins) and 2,942 edges (interactions) for the blue module, 204 nodes and 1,085 edges for the yellow module, 183 nodes and 982 edges for the red module, 131 nodes, and 1,905 edges for the purple module, 121 nodes and 716 edges for the greenyellow module, and 91 nodes and 345 edges for the salmon module indicating high connection density of proteins encoded by the genes of these modules. Figure 8 represents the PPI network of the purple module as the nonpreserved and potential biologically BRD-related module. Additionally, based on the PPI networks obtained by hub genes, a total number of 48, 51, 48, 49, 27, and 19 hub–hub genes were found in the blue, yellow, red, purple, greenyellow, and salmon modules, which can be potential biomarkers and candidate disease genes in the etiology and the diagnostics of BRD (Table 2). PPI networks of significant highly correlated modules (identified by MTRs) and nonpreserved modules (identified by MP) that were biologically associated with BRD are available in Supplementary Figure S1.

**TABLE 2:** List of the hub–hub genes of the nonpreserved and biologically BRD-related modules that were identified by module preservation analysis.

Modules					
Blue	Greenyellow	Purple	Red	Salmon	Yellow
LAMP1	STAG2	ISG15	RAB11FIP2	IL2RB	C5AR1
LAMP2	PIK3CA	STAT1	TNF	KLRK1	IL1B
TLR4	SMURF2	IFIH1	MAU2	PRF1	UBA52
PSAP	PTEN	RTP4	UNKL	ITGAL	PTAFR
ANXA5	ADAM10	USP18	TAF1	CCL5	IL15
C3	TSPAN13	DDX58	NR2C2	CCR5	GRB2
PSEN1	SNX27	IRF7	LCK	GZMA	CD68
RAP1B	KLHL11	MX1	CD2	NCR1	PLEK
CTSB	PIP5K1B	PARP9	ARIH2	RUNX3	SOCS3
STOM	SOS2	DHX58	ZFYVE20	S1PR5	NFKBIA
ACTR2	SNX13	IFI35	TSC1	CX3CR1	MYD88
BECN1	MGAT4A	RSAD2	ITK	NKG7	ANXA1
CAT	YES1	IFI44	SMC5	IL12RB2	FCAR
NPC2	GSK3B	IFI44L	LCP2	GZMB	CYTH4
CD86	SGK3	UBA7	PHF8	CCR8	CRKL
ATG7	UBE2R2	UBE2L6	JAK3	PDCD1	HECW2
VPS35	TMEM30A	EIF2AK2	RIC1	TRPM2	RAF1
PRCP	HECTD1	IRF9	SETDB1	TMEM63A	IL18RAP
RAB1A	TSPAN33	ISG20	FMR1	GZMH	GADD45B
RAB5A	RNF217	MX2	PLCG1	—	FAS
GAA	ROCK1	XAF1	CHD3	—	CNR2
MGST1	TAB2	PARP14	FBXO4	—	CCR1
CCR2	PDP1	PARP12	FBXL20	—	HCAR3
CD59	WAPAL	IFIT5	PIK3CD	—	SNX18
CTSC	WAC	STAT2	FBXO21	—	SGK1
ACTR1A	PI4K2B	TRIM21	TIA1	—	IFNAR2
TLR7	SERPINE2	OAS1X	TNRC6A	—	VNN2
LYZ	—	HERC6	TLR3	—	DDIT3
GM2A	—	CMPK2	POGZ	—	WDFY3
CST3	—	ZBP1	PDPR	—	IFNGR2
TNFRSF1B	—	DTX3L	CRAMP1L	—	PPP2R5A
CAPZA1	—	ZNFX1	RERE	—	SELL
RAB6A	—	IFITM3	RAB11FIP4	—	KDM4B
ARF1	—	RNASEL	CD6	—	NCF1
FTL	—	SAMD9	ZC3H11A	—	LOC407171
APLP2	—	GBP4	TNFRSF10D	—	ARRDC4
PECAM1	—	TRIM25	UBP1	—	TARM1
ATF6	—	MB21D1	SLC37A3	—	VASP
CSF2RB	—	OAS1Y	IKBKE	—	SLC2A3
RAB18	—	HERC5	STAT5A	—	BST1
SPTLC1	—	ADAR	NAA16	—	MCL1
KTN1	—	GBP7	ZBTB43	—	NFAM1
SHISA5	—	OAS2	CAMSAP1	—	TNIP1
MOSPD2	—	IFI6	TWF1	—	GADD45A
CD163	—	FOXS1	ABI2	—	NUDT3
ATP6V1A	—	PML	VAMP5	—	MEFV
IFNAR1	—	CD53	CDC7	—	MXD1
CAPN2	—	CHMP5	UPB1	—	LONRF3
—	—	GPR97	—	—	KDM6B
—	—	—	—	—	ICAM3
—	—	—	—	—	TCN1

## Discussion

BRD is a multifactorial disease that results from the interaction of environmental stressors and infectious agents of BRDC (Gagea et al., 2006). BRDC includes the viral pathogens such as bovine respiratory syncytial virus (BRSV), bovine parainfluenza type 3 virus (BPIV-3), bovine viral diarrhea virus (BVDV), bovine coronavirus (BCV), and bovine herpes virus type 1 (BHV-1) that affect upper respiratory system and also contain the bacterial pathogens *Trueperella pyogenes*, *Mycoplasma bovis*, *Pasteurella multocida*, *Histophilus somni*, *Bibersteinia trehalosi*, and *Mannheimia haemolytica*, which can affect the lower respiratory system (Caswell, 2014; Kirchhoff et al., 2014). Despite numerous studies, BRD is still the most common disease and the leading cause of morbidity and mortality in the cattle industry (Taylor et al., 2010). Understanding the molecular mechanisms involved in the bovine immune response to BRD is necessary given the persistence of the disease in recent years. Combining high-throughput technologies with various computational methods based on the network approach, can provide an exceptional opportunity to better understand the pathological processes of diseases and the molecular mechanisms of the host immune responses (Kadarmideen and Watson-Haigh, 2012). In this study, we combined gene expression matrix obtained by RNA-seq data analysis with two co-expression network-based methods of WGCNA, module–trait relationships, and module preservation analysis, to identify potential gene modules and candidate genes involved in molecular processes induced by BRD.

### Module–Trait Relationships Analysis

Module–trait relationship analysis identified four significant highly correlated modules including turquoise, purple, blue, and brown with clinical traits of BRD. Functional enrichment analysis indicated that three of the four (75%) identified modules including blue, purple, and turquoise modules had significant enriched terms and pathways. This shows the power and high accuracy of signed networks in separating the modules from each other and identifying more significantly enriched terms or pathways in the modules. The significant enriched terms in the blue module were mostly related to basic cellular activities including “tRNA transport,” “transcription DNA-templated,” “DNA metabolism,” “macromolecule biosynthetic,” and “cell cycle.” On the other hand, the turquoise and purple modules had many biological processes and KEGG pathways closely related to the BRD mechanisms. So, our focus on the turquoise and purple modules (identified by MTRs method) as significant highly correlated and key biologically related modules to BRD.

Coregulated genes in the turquoise module were closely related to the mechanisms of the innate immune system as the first defense line against BRD and this shows the great importance of this module during BRD development. We also found a number of enriched terms related to the adaptive immune system in this module. After infection with various viral or bacterial pathogens, extensive complex interactions begin between the host and the pathogen (Kumar et al., 2011). Pathogens produce various molecules known as pathogen-associated molecular pattern (PAMPs) to continue their life cycle and pathogenic activity (Janeway and Medzhitov, 2002; Tang et al., 2012). These PAMPs are recognized by pattern recognition receptors (PRRs) in the host, which are proteins expressed by key innate immune system cells such as neutrophils, macrophages, monocytes, dendritic cells, and epithelial cells (Medzhitov, 2007; Yuki and Koutsogiannaki, 2021). Upon recognition of PAMPs by PRRs, a cascade of signaling pathways is induced, leading to an inflammatory response and subsequent rapid response of the innate immune system to eliminate the pathogens (Lee and Kim, 2007; Kawai and Akira, 2010). In this regard, the turquoise module had important KEGG signaling pathways as well as their downstream biological processes associated with PRRs and inflammatory response including “Toll-like receptor signaling pathway,” “MyD88-dependent Toll-like receptor signaling pathway,” “TRIF-dependent Toll-like receptor signaling pathway,” “C-type lectin receptor signaling pathway,” “NOD-like receptor signaling pathway,” “NF-kappa B signaling pathway,” and “MAPK signaling pathway.”

Toll-like receptors (TLRs) are the most important signaling maker PRRs and act as the primary sensors of pathogens (Iwasaki and Medzhitov, 2004; Akira et al., 2006). The Toll-like receptor signaling pathway is activated through the recognition of PAMPs by membrane and cytoplasmic TLRs (Kumar et al., 2009; Blasius and Beutler, 2010; Heidarzadeh et al., 2020). The TLR signaling pathway is divided into two distinct pathways, including the MyD88-dependent and the TRIF-dependent signaling pathway, depending on the type of TLR sensitized and subsequently equipped adapters (Takeuchi and Akira, 2010). All TLRs family (*TLR1* to *10*), except *TLR3*, activate the MyD88-dependent Toll-like receptor signaling pathway, which activates the NF-kappa B signaling pathway and MAPK signaling pathway to produce proinflammatory cytokines and chemokines (Takeda and Akira, 2005; Lin et al., 2010; Kawasaki and Kawai, 2014). On the other hand, *TLR3* as well as *TLR4* activate the TRIF-dependent Toll-like receptor signaling pathway, which leads to induce production of proinflammatory cytokines and type I interferons by activating *NF-κB* and *IRF3/IRF7* transcription factors (Takeda and Akira, 2005; Lin et al., 2010; Kawasaki and Kawai, 2014). Various studies in cattle diseases such as bovine tuberculosis (Nalpas et al., 2015), Johne’s disease (Ferwerda et al., 2007; Wang et al., 2019), endometritis (Turner et al., 2014), and mastitis (Luoreng et al., 2018; Islam et al., 2020) have reported the TLR-signaling pathway being induced during these diseases. A previous study also reported that the Toll-like receptor signaling pathway was activated during different challenges with a group of BRDC, including BoHV-1, BRSV, BVDV, *Mannheimia haemolytica*, and *Pasteurella multocida* (Tizioto et al., 2015).

C-type lectin receptor signaling pathway is another pattern recognition receptor related pathway that is activated by CLRs membrane receptors by identifying different carbohydrates such as mannose, glucan, and fucose in viruses, bacteria, and fungi and activates MAP kinases, the transcription factor *NF-AT*, and *NF-κB* that eventually induces the production of proinflammatory cytokines (Geijtenbeek and Gringhuis, 2009; Takeuchi and Akira, 2010). Recent studies have examined the importance of C-type lectin receptors in human infectious diseases (Lugo-Villarino et al., 2018; Zhao et al., 2019). NOD-like receptors are cytosolic receptors that can detect a wide range of bacteria, viruses, and other pathogens that enter the cytoplasm (Franchi et al., 2009). These receptors, like Toll-like receptors activate the NF-kappa B and MAPK signaling pathways, regulate the production of inflammatory cytokines such as *IL-1β*, and can also induce apoptosis (Chen et al., 2009; Banse et al., 2013).

The NF-kappa B signaling pathway is a key pathway that acts as a major mediator in inflammatory responses. Activation of the *NF-κB* transcription factor induces the transcription of many genes that encode proinflammatory cytokines and chemokines such as *IL-1β*, *IL-6*, *TNF-α*, *IL-12p40*, and *cyclooxygenase-2* (Oeckinghaus and Ghosh, 2009; Liu et al., 2017). In addition, a study investigated the effect of SH protein expressed by the BRSV genome on the lack of *NF-κB* phosphorylation in the host, which reduces the production of proinflammatory cytokines and thus modulates the immune system (Pollock et al., 2017). The MAPK signaling pathway is another key mediator pathway during inflammation that regulates cytokine production by phosphorylating and activating certain kinases (Salojin and Oravecz, 2007). Several transcriptomics and proteomics studies have reported MAPK signaling pathway activity during BRD (Tizioto et al., 2015; Behura et al., 2017; Liyang et al., 2021).

Furthermore, other important immune-related terms identified in the turquoise module include “JAK-STAT signaling pathway,” “TNF signaling pathway,” “PI3K-Akt signaling pathway,” “regulation of interferon-gamma production,” and “positive regulation of interleukin-8 secretion.” The JAK-STAT signaling pathway is one of the most important intracellular signaling pathways that regulates communication between cytokine transmembrane receptors and the nucleus and is involved in many biological processes in the body, such as immune regulation, cell differentiation/proliferation, apoptosis, and keep homeostasis in inflammatory conditions (O'Shea et al., 2012; O'Shea et al., 2015; Xin et al., 2020). This pathway, mediates cytokine responses through binding of cytokines such as *IL-6/12/17/23*, as well as type I (alpha and beta), and II (gamma) interferons to their respective receptors at the cell surface and activation of STATs transcription factors to regulate their target genes in the nucleus (Charles Jay and Eric, 2009). Interferons by activating this pathway, can cause antiviral conditions (Horvath, 2004b; a). BoHV-1, as an infectious agent of BRDC, has the ability to suppress the host immune system by expressing the *UL41* gene and subsequently increase its viral replication. The *UL41* blocks the JAK-STAT signaling pathway by suppressing *STAT1* expression. Thus, this virus has the ability to deal with the antiviral condition resulting from the JAK-STAT signaling pathway (Ma et al., 2019). On the other hand, Ma et al. (2020) reported that *Bta-miR-2890*, by directly targeting BoHV-1 *UL41*, increases *STAT1* and *JAK1* expression and, thus, opens the JAK-STAT signaling pathway as well as prevent viral replication. This suggests the importance of JAK-STAT signaling pathway during viral infections.

The PI3K–Akt signaling pathway is a key pathway in all mammalian cells that is involved in several processes, such as cell growth, migration, proliferation, and metabolism as well as the role of this pathway in regulatory T-cell development and memory CD8 T-cell differentiation, has been reported (Kim and Suresh, 2013; Pompura and Dominguez-Villar, 2018). It has also been suggested that some genes in the PI3K–Akt signaling pathway may play an important role in eliciting downstream cascades in lung lesions during BRD (Behura et al., 2017). Tumor necrosis factor (TNF) is a proinflammatory cytokine that is mainly secreted by macrophages and alerts other cells during the inflammatory response. TNF is also known to be a major regulator of the production of proinflammatory cytokines and has been considered as a therapeutic target for the treatment of some inflammatory diseases such as rheumatoid arthritis and inflammatory disease (Liu, 2005; Parameswaran and Patial, 2010). The TNF signaling pathway plays an important role in the control of inflammation, immunity, and cell survival (Rana et al., 2019). It has been shown that one of the strategies for bacterial survival and immunosuppression by *Mycoplasma bovis* is to inhibit *TNF-α* and interferon-gamma production (Mulongo et al., 2014).

Interleukin-8 is a chemokine, that is expressed in various cells especially in macrophages. *IL8* is responsible for the induction of chemotaxis and causes guided migration of neutrophils to the site of infection (Remick, 2005). An increase in *IL8* expression has been observed in challenges with BRSV and *Mannheimia haemolytica* as well as a significant association between increased expressions of *IL8* with lung lesions. Therefore, it has been suggested that *IL8* antagonist drugs be used to prevent inflammatory lung lesions (Caswell et al., 1998; Malazdrewich et al., 2001; Singh et al., 2011; Redondo et al., 2014).

In response to signaling proinflammatory cytokines and chemokine such as *IL8*, neutrophils are the first cells to migrate from the blood to the infection site (Kolaczkowska and Kubes, 2013). These cells play an important role in killing extracellular pathogens through phagocytosis (Nordenfelt and Tapper, 2011; DeLeo and Allen, 2020). Neutrophil-related biological processes and KEGG pathways in the turquoise module included “neutrophil mediated immunity,” “neutrophil degranulation,” “neutrophil activation involved in immune response,” and “neutrophil extracellular trap formation.” However, neutrophils play an important role in the pathogenesis of BRD by destruction and damaging lung tissue during infection (McGill and Sacco, 2020). Moreover, neutrophils release their nuclear DNA and related proteins in the extracellular environment through NETosis, a unique form of cell death that leads to the formation of neutrophil extracellular traps (McGill and Sacco, 2020). NETs trap and kill bacteria, fungi, viruses, and parasites. In addition to their antimicrobial role, NETs can also play a role in the pathogenesis of inflammatory diseases (Papayannopoulos, 2018). Furthermore, evidence suggests that NETs play a role in the host defense in response to *Mannheimia haemolytica* and *Histophilus somni* infection (Aulik et al., 2010; Hellenbrand et al., 2013). The findings also indicate that *Mycoplasma bovis*, through its endonucleases, is able to digest NETs and escape the immune system (Gondaira et al., 2017). However, Cortjens et al. (2016) showed that NETs has the ability to trap respiratory syncytial virus (RSV), but their excessive accumulation leads to airway obstruction, which can contribute to RSV pathogenesis (Cortjens et al., 2016).

We also identified several microbicidal mechanisms, including “Phagocytosis,” “Fc gamma R-mediated phagocytosis,” “Phagosome,” “Lysosome,” and “phagosome maturation” in the turquoise module. In the immune system of multicellular organisms, phagocytosis is a cellular process for the elimination of pathogens and dead cell debris, and is an essential for maintaining tissue homeostasis (Flannagan et al., 2012). Fc gamma R (FcγR) receptors are glycoproteins found on the surface of immune cells such as neutrophils, macrophages, and natural killer cells that stimulate phagocytosis through antigen-binding IgG antibodies (Rosales, 2017). Phagocytosis involves several stages. After ingestion of pathogens or foreign particles by immune cells, the ingested particles become specialized vacuoles and distinct organelles called phagosomes. During the stage called phagosome maturation, the lysosome fuses with the phagosome membrane, and its contents are poured into the phagosome, and an organelle called phagolysosome is formed. This newly formed organelle contains enzymes that can break down the digested particles (Canton, 2014; Levin et al., 2016; Uribe-Querol and Rosales, 2020). Because of the importance of phagocytosis pathway in inducing an innate and adaptive immune response, pathogens use specific strategies to control and suppress phagocytes (Srikumaran et al., 2007). *Mannheimia haemolytica* and *Pasteurella multocida* use their toxins and extracellular components to kill phagocytes, thus preventing phagocytosis and subsequently releasing the reactive oxygen metabolite contents of the phagocytes, which exacerbates pulmonary inflammation (CUSACK et al., 2003). Research also shows that *Mycoplasma bovis* has the ability to survive long-term in necrotic lung lesions and phagocytic cells by overcoming phagocytosis (Kleinschmidt et al., 2013). Moreover, virulent isolates of *Histophilus somni* have the ability to survive in phagocytic cells by interfering with phagosome–lysosome maturation (Pan et al., 2018). In addition to the bacterial agents of the BRDC, viruses such as BVDV, PIV3, and BRSV have the ability to inhibit phagocytosis by macrophages (Bell et al., 2021).

The turquoise module also identified some of the major pathways associated with programmed cell death, including “Apoptosis” and “Necroptosis.” Apoptosis is the first type of programmed cell death that clears cells infected with pathogens (especially viruses) to prevent them from replication and spread (Amarante-Mendes et al., 2018). For example, a previous study showed that apoptosis of BRSV-infected epithelial bronchial cells is an effective way to clear the virus (Viuff et al., 2002) as well as it has been suggested that apoptosis may play a role in modulating airway inflammation during BRSV infection (Cristina et al., 2001). On the other hand, the BHV-1 uses the products of the *LR* gene to prevent apoptosis of infected cells and can therefore proliferate sufficiently in infected cells (Geiser and Jones, 2005; Srikumaran et al., 2007). Moreover, BHV-1 inhibits the efficient immune response by infecting CD41^+^ T-cells and inducing apoptosis in them (Jones and Chowdhury, 2010). Bacterial pathogens also have the ability to survive and manipulate intracellular mechanisms of host cells to escape the immune system. A recent study showed that *Mycoplasma bovis* has the ability to inhibit the apoptosis of infected bovine alveolar macrophages by increasing the expression of several anti-apoptotic genes (Maina et al., 2019). Necroptosis is a programmed form of inflammatory cell death (necrosis) that actively causes cell death of infected cells or the propagation of danger signals to stimulate the immune system. However, uncontrolled necroptosis may lead to the pathogenesis of inflammatory diseases. For instance, the findings show that RSV, through the activation of necroptosis, lead to neutrophilic cell death (Muraro et al., 2018). Bedient et al. (2020) showed that the RSV causes cell death of alveolar macrophages through various cell death mechanisms such as necroptosis, pyroptosis, and apoptosis, which can contribute to the pathogenesis of the RSV and exacerbate inflammation in the lungs (Bedient et al., 2020).

Interestingly, we identified the terms “regulation of nitric oxide biosynthetic process,” “positive regulation of nitric oxide metabolic process,” and “positive regulation of nitric oxide biosynthetic process” in the turquoise module. Nitric oxide (NO) is a natural molecule with antimicrobial properties that is produced by most mammalian cells (Crepieux et al., 2016). Several studies have shown the antibacterial and antiviral properties of NO during BRD, and it has also been demonstrated that NO therapy, as a non-antibiotic-based treatment can be a safe and effective method to control BRD (Regev-Shoshani et al., 2013; Regev-Shoshani et al., 2014). Another study demonstrated that NO reduced levels of proinflammatory cytokines, such as *IL-1β* and *TNF*, thereby limiting inflammation during BRD. On the other hand, NO increases the ability of the host to detect pathogens by increasing the expression of *TLR4* gene (Sheridan et al., 2016).

We also identified some pathways in the turquoise module associated with the adaptive immune system such as “B-cell receptor signaling pathway”, “positive regulation of activated T-cell proliferation,” “Th1/Th2 cell differentiation,” and “Th17 cell differentiation.” B cells are vital cells for the humoral response, and research shows that the B-cell receptor signaling pathway is one of the most important pathways in the immune system of animals to respond to infection with viral agents of BRDC (Tizioto et al., 2015). T cells are essential cells for cell-mediated immunity and include several subtypes that play a variety of roles, including killing virus-infected cells, secreting interferon-gamma, and other cytokines (Platt et al., 2006). The importance and application of helper and cytotoxic T cells in viral clearance in the first and second challenges with BVDV have been investigated. The results show that these T cells in the second challenge with BVDV have the ability to quickly clear the virus (Silflow et al., 2005). Furthermore, T cells have been reported to be critical for the response to BRSV infection (Gaddum et al., 2003).

Intramodular hub genes (especially hub–hub genes) are highly correlated with the biological function of the module. In this regard, hub–hub genes identified in the turquoise module, such as *FN1*, *MAPK14*, *PSEN1* (Neupane et al., 2018), *IL-1β*, *IL-18* (Taylor et al., 2014), *MYD88* (Dubbert et al., 2013), *SOD2* (Hofstetter and Sacco, 2020), *CTSD* (Gray et al., 2019), *JAK2* (Amat et al., 2019; Chao et al., 2019), *CD68* (Lee et al., 2009), and *TLR2* (Mariotti et al., 2009; Tizioto et al., 2015) have been reported as important genes in previous BRD studies. *SOCS3* hub–hub gene was another key gene identified in the turquoise module. This gene blocks both the production and signal transduction of type I and II interferons by disrupting the JAK/STAT signaling pathway (Akhtar and Benveniste, 2011). Several studies have shown that viral agents of BRDC disrupt interferon-dependent antiviral responses in the host by inducing the expression of *SOCS1* and *SOCS3* and subsequently provide a suitable condition for their survival and proliferation (Akhtar and Benveniste, 2011; Ye et al., 2015; Zheng et al., 2015; Alkheraif et al., 2017; Salem et al., 2019). These findings indicate the importance of the *SOCS3* gene as a therapeutic target for infections caused by BRDC viral agents.

Moreover, other important hub–hub genes in the turquoise module included *IL10* and *TLR4*. Interleukin-10 (*IL10*) is an anti-inflammatory cytokine that inhibits inflammatory responses initiated by proinflammatory cytokines and subsequently regulates inflammation (Pestka et al., 2004). Therefore, some studies show that increasing the expression of *IL10* regulates the inflammatory response during BRD (Molina et al., 2014; Gondaira et al., 2015; Rodríguez et al., 2015). On the other hand, Risalde et al. (2011) showed that BHV-1 secondary infection in BVDV-infected cows suppressed *IL10* expression, which leads to exacerbate the inflammatory response and more severe clinical lesions (Risalde et al., 2011). Furthermore, failure in upregulation of *IL10* expression level due to weaning and transport is associated with a doubling of BRD mortality (Hodgson et al., 2005). *TLR4* is one of the most important cell surface PRRs that induces an inflammatory response in response to lipopolysaccharide (LPS) derived from Gram-negative bacteria by activation of different signaling pathways (Ciesielska et al., 2021). However, overexpression and abnormal activation of *TLR4* leads to chronic and acute inflammatory disorders such as endotoxemia and sepsis in human and equine (Werners and Bryant, 2012; Kuzmich et al., 2017). Also, due to the key role that *TLR4* plays in activating the signaling pathways that lead to the secretion of proinflammatory cytokine, this gene has been suggested as a very attractive therapeutic target for inflammatory diseases, such as sepsis in human and equine (Werners and Bryant, 2012; Kuzmich et al., 2017). Moreover, a significant correlation has been reported between increased *TLR4* expression level and increased mortality during BRD (Hodgson et al., 2012). Therefore, these results indicate a key role for *IL10* and *TLR4* during BRD that can be further explored as important targets.

Among the detected TFs, *STAT3* is one of the most important hub–hub TFs identified in the turquoise module. Signal transducer and activator of transcription 3 (*STAT3*) is a transcription factor belonging to the STAT protein family that regulates various biological activities such as apoptosis, angiogenesis, differentiation, cell proliferation, inflammation, and the immune response (Furtek et al., 2016). In different studies, *STAT3* has been suggested as a key gene involved in various bovine diseases (Jaslow et al., 2018; Villaseñor et al., 2019; Bakhtiarizadeh et al., 2020). Moreover, several transcriptomic studies have identified *STAT3* among the top DEGs in response to infection with agents of BRDC, indicating its important role during BRD (Wu et al., 2017; Chao et al., 2019).

Several functional terms identified in the purple module included “Natural killer cell-mediated cytotoxicity,” “T-cell receptor signaling pathway,” “cellular defense response,” “Chemokine signaling pathway,” and “T-cell activation.” Interestingly, in addition to the antiviral activity of T cells that have been discussed above, the T-cell receptor signaling pathway has also been observed in challenges with *Mannheimia haemolytica*, which indicates the importance and participation of T cells in responding to different types of pathogens (Tizioto et al., 2015). In agreement with the previous studies, *PRF1* (Johnston et al., 2021a), *LCK* (Smirnova et al., 2009), *NCR1* (Osman and Griebel, 2017), *CCL5* (N’jai et al., 2013), *CD8A* (Knapek et al., 2020; Lebedev et al., 2021), *CCR8* (Lopez et al., 2020), *CX3CR1* (Salem et al., 2019), and *TBX21* hub–hub TF (Johnston et al., 2019) were identified as highly connected genes in the purple module and have been reported as immune-related genes during BRD. For instance, the *PRF1* hub–hub gene is an important gene that encodes the perforin-1, which is present in cytotoxic T-lymphocytes (CTLs) and natural killer cells (NK cells) and is involved in cytolysis and the regulation of the immune system. This gene has been suggested by Johnston et al. (2021a) as one of the biomarkers for diagnosis of subclinical BRD from blood samples.

### Module Preservation Analysis

Module preservation analysis and functional enrichment showed that six modules (blue, greenyellow, purple, red, salmon, and yellow) first changed their connectivity pattern and network density due to BRD and second, were biologically related to the BRD development. As expected, highly preserved and semipreserved modules were more active in basic and general cellular activities such as “Ribosome,” “rRNA processing,” “DNA packaging,” “peptide biosynthetic process,” and “translation.” Therefore, based on highly preserved and semipreserved modules, it is not possible to explain the molecular mechanisms involved in BRD. On the other hand, the six mentioned nonpreserved modules were closely related to the immune system and the pathogenesis of BRD.

Functional terms in the blue module were mostly related to the innate immune system, including “lysosome,” “phagosome,” “Fc gamma R-mediated phagocytosis,” “leukocyte transendothelial migration,” “Toll-like receptor signaling pathway,” “Chemokine signaling pathway,” “neutrophil activation involved in immune response,” “activation of MAPK activity,” and “positive regulation of NF-kappaB import into nucleus.” The leukocyte transendothelial migration is one of the important steps of the innate immune system in triggering the inflammatory immune response and the migration of the first immune response cells such as neutrophils to the sites of infection (Getter et al., 2019; Bakhtiarizadeh et al., 2020) which that chemokines control cellular responses at inflammatory sites through this pathway (Krishnan et al., 2004). In agreement with similar BRD studies (Tizioto et al., 2015; Behura et al., 2017; Johnston et al., 2021c; Lebedev et al., 2021), the leukocyte transendothelial migration was identified as one of the important pathways of the immune system in the blue module. Additionally, some of the hub–hub genes identified in the blue module have also been reported in the previous BRD studies, including *TLR4* (Scott et al., 2021), *ANXA5* (Mitchell et al., 2008), *C3*, *PSEN1* (Neupane et al., 2018), *CTSB*, *CD59*, *FTL* (Nilson et al., 2020), *CAT* (Joshi et al., 2018), *TLR7*, *CD86* (Palomares et al., 2014), *ATG7* (Lipkin et al., 2016), and *IFNAR1* (Amat et al., 2019). For example, stresses from weaning, transport, and commingling reduced the expression level of the *ANXA5* (hub–hub gene) and, thus, lead to an increase in apoptotic cells in the lungs or epithelial lining fluid, which can increase the susceptibility of cattle to a primary infection (Mitchell et al., 2008).

Coexpressed genes in the greenyellow module were enriched in KEGG pathways and biological process such as “T-cell receptor signaling pathway,” “focal adhesion,” “leukocyte transendothelial migration,” and “regulation of cell motility.” These pathways have also been observed in previous transcriptomic studies in response to infection with the agents of BRDC (Tizioto et al., 2015; Behura et al., 2017). Among the hub–hub genes identified in the greenyellow module, the *PTEN* gene plays an important role in the pathogenesis of BRD. Research has shown that *MicroRNA-26b* induces the NF-kB signaling pathway by directly targeting *PTEN*, which exacerbates inflammation in the lungs during infection with Gram-negative bacteria (Zhang et al., 2015). Moreover, other hub–hub genes in the greenyellow module including *ADAM10* (Neupane et al., 2018), *MGAT4A* (Johnston et al., 2021b), and *GSK3B* (Chao et al., 2019) were identified as important genes involved in BRD.

Functional enrichment analysis revealed that the purple module was enriched in the “NOD-like receptor signaling pathway,” “C-type lectin receptor signaling pathway,” “RIG-I-like receptor signaling pathway,” and “necroptosis” KEGG pathways as well as “inflammatory response,” “type I interferon signaling pathway,” “positive regulation of type I interferon production,” “interferon-gamma-mediated signaling pathway,” and “cellular response to interferon-gamma” biological processes. RIG-I-like receptors are important cytoplasmic PRRs that detect intracellular viruses through their genomic RNA (Takeuchi and Akira, 2010). Recognition of PAMPs by RIG-I-like receptors initiates the RIG-I-like receptor signaling pathway, which activates *NF-κB* and *IRF3/IRF7* transcription factors which subsequently lead to the production of proinflammatory cytokines and type I interferons (Kumar et al., 2011). Type I (IFN-α and IFN-β) and II (IFN-γ) interferons are cytokines that are the first line of defense against viral infections that play a key role in the development of antiviral states during the immune response (Platanias, 2005). Type I interferons are polypeptides that are secreted from virus-infected cells and activate antimicrobial states in infected cells and healthy neighboring cells to prevent the proliferation and growth of infectious agents, especially viruses. They also promote antigen presentation, increase the activity of natural killer cells, and activate the adaptive immune system (Ivashkiv and Donlin, 2014). Furthermore, type II interferon, whose major producers include natural killer cells, T cells, macrophages, dendritic cells, and B cells play a key role in regulating many protective functions such as inducing antiviral states, enhancing antimicrobial functions, increasing leukocyte trafficking, effect on cell proliferation, and apoptosis (Kak et al., 2018).

Several recent studies have shown that the type I interferon signaling pathway has been identified as a key pathway in cows with BRD atfeedlot entry, which indicates that animals show antiviral responses at the entry stage (Sun et al., 2020; Johnston et al., 2021a; Scott et al., 2021). Interestingly, some of the important hub–hub genes, which are involved in anti-viral interferon response were identified in the purple module, such as *IFI6*, *ISG15*, *MX1*, *OAS2*, *IFIH1*, *DDX58*, *DHX58*, *RSAD2*, *IFI44*, *IFI44L*, *EIF2AK2*, *ISG20*, *MX2*, *IFIT5*, *IFITM3*, *OAS1Y*, and *HERC5*, have been suggested by these studies as potential biomarkers for diagnostic and prediction of subclinical BRD at early stage of infection (Sun et al., 2020; Johnston et al., 2021a; Scott et al., 2021). Additionally, some of the key hub–hub TFs that regulate the expression of coexpressed genes in this module, and play critical roles in interferon antiviral responses during BRD, were included *IRF9*, *STAT1*, *STAT2* (Sun et al., 2020), and IRF7 (Johnston et al., 2019). Type I interferons activate the IFN-stimulated gene factor 3 (*ISGF3*) complex during JAK/STAT signaling pathway. This complex consists of three transcription factors *STAT1*, *STAT2*, and IFN-regulatory factor 9 (*IRF9*), which induce the expression of antiviral genes (Ivashkiv and Donlin, 2014). In addition to type I interferons (IFN-α and IFN-β), type II interferons (IFN-γ) also cause the formation of STAT1-STAT1 homodimers, which, following phosphorylation and after being transferred to the nucleus, induce the expression of antiviral genes (Platanias, 2005; Kak et al., 2018). Besides, several studies have demonstrated that cytoplasmic localized infected cell protein 0 (*bICP0*) encoded by the BHV-1 (a viral agent of BRDC), through interaction with *IRF7*, disrupts the activity of the IFN-β promoter (Saira et al., 2007; da Silva et al., 2011; Jones, 2019). Moreover, one study demonstrated that BPIV-3 had a negative effect on the JAK/STAT signaling pathway by reducing phosphorylation of *STAT1*, thereby inhibiting the production of antiviral molecules (Eberle et al., 2016). These explain why the *STAT1*, *STAT2*, *IRF9*, and *IRF7* transcription factors are highlighted in this module as very important regulators. Other members of the purple module, including *USP18*, *OAS1X*, *CMPK2*, *GBP4* (Nilson et al., 2020), *IFI35* (Sun et al., 2020), *PARP14* (Oguejiofor et al., 2015), *RTP4* (Johnston et al., 2019), and *TRIM21* (Quick et al., 2020), have also been observed in different BRD studies that may play an important role in the immune system in response to BRDC agents.

In agreement with the previous studies, functional terms including “VEGF signaling pathway,” “T-cell receptor signaling pathway” (Tizioto et al., 2015), and “C-type lectin receptor signaling pathway” as well as hub–hub genes including *LCK* (Smirnova et al., 2009), *TLR3* (Marin et al., 2016), *TIA1* (Johnston et al., 2021b), *TNF* (El-Deeb et al., 2020), and *STAT5A* hub–hub TF (Lin et al., 2015) demonstrate the importance of the red module during BRD. Furthermore, the salmon module was mainly enriched in “cellular defense response,” “regulation of immune response,” “leukocyte cell–cell adhesion,” and “natural killer cell-mediated cytotoxicity.” Recent studies have demonstrated that some of the hub–hub genes in the salmon module, such as *CCR5* (Salem et al., 2019), *CCR8* (Amat et al., 2019; Lopez et al., 2020), *CX3CR1* (Scott et al., 2021), *ITGAL*, *IL12RB2* (Neupane et al., 2018), *NCR1* (Osman and Griebel, 2017), *CCL5* (N’jai et al., 2013), and *PRF1* (Johnston et al., 2021a) tend to participate in BRD. Additionally, *GZMB* gene, which plays a major role in stimulating cytotoxic T-cell responses and limiting virus replication in the host (Jiminez et al., 2021), was found among hub–hub genes in the salmon module. Granzyme B-protein, which is encoded by this gene, is an important serine protease that is expressed in cytotoxic T-lymphocytes (CTL) and natural killer (NK) cells and kills viral infected cells through apoptosis (Xu et al., 2018). *GZMB* was the most highly upregulated gene in the bronchial lymph node in response to BRSV infection, indicating a close relationship between this gene and the response to viral infection (Johnston et al., 2019). This gene has also been found to be among the DEGs with the highest expression in animals that are resistant to BRD (Scott et al., 2020). The *GZMB* gene is likely to play an important role in the host defense against BRSV infection and maybe other BRDC viral agents, and changes in this gene are critical for BRD resistance and susceptibility (Johnston et al., 2019), as shown, the presence of polymorphisms in the *GZMB* gene in mice caused the cytotoxic T cells to lose their ability to kill viral infected cells (Andoniou et al., 2014).

The yellow module showed that its genes were enriched in some important biological processes and KEGG pathways associated with BRD such as “neutrophil activation involved in immune response,” “regulation of inflammatory response,” “positive regulation of T-cell proliferation,” “MAPK signaling pathway,” “NF-kappa B signaling pathway,” “apoptosis,” “TNF signaling pathway,” “JAK-STAT signaling pathway,” and “Cytokine-cytokine receptor interaction.” Furthermore, examination of the relationship between the hub–hub genes of this module and BRD showed that many of these genes, such as *IL1B* (Behura et al., 2017), *IL15* (Leach et al., 2012; Amat et al., 2019), *CD68* (Buchenau et al., 2010; Hermeyer et al., 2011), *SOCS3* (Ye et al., 2015; Zheng et al., 2015), *NFKBIA*, *RAF1*, *SGK1* (Chao et al., 2019), *ANXA1* (Mitchell et al., 2008), *MYD88* (Dubbert et al., 2013), *FAS* (Xu et al., 2012), *CCR1* (Lindholm-Perry et al., 2018), *IFNAR2* (Schlender et al., 2000), *NFAM1*, *NUDT3* (Johnston et al., 2021b), and *SLC2A3* (N’jai et al., 2013) as well as *DDIT3* hub–hub TF (Wang S. et al., 2020) have important effects on the interaction between the host and the pathogen. For example, stressful stimuli directly increase the expression level of the *SGK1* (hub–hub gene), and consequently an upregulation in the expression of this gene leads to stimulate BoHV-1 and HSV-1 replication (Kook and Jones, 2016; Zhu et al., 2017). Therefore, the use of SGK inhibitors may be a suitable strategy to reduce BoHV-1 and HSV-1 replication (Kook and Jones, 2016).

These findings demonstrate the relevance of the mentioned modules as well as their genes, especially hub–hub genes and TFs as important candidates in the development of BRD, helping us to better understand the molecular mechanisms responsible for the immune response to BRD. Further researches are needed to more closely examine the biological behavior and functions of these modules and their genes during BRD.

## Conclusion

Given that BRD is the main cause of morbidity and mortality in beef and dairy cattle and has a potential impact on economic losses in the livestock industry, a systems biology approach was used to further investigate the molecular mechanisms of BRD as well as to identify diagnosis biomarkers and therapeutic targets for BRD. In this study by using WGCNA distinct methods (MTRs and MP) and functional enrichment analysis, we identified eight candidate modules that are involved in the immune response and BRD pathogenesis. It is noteworthy that both WGCNA methods showed a similar ability to identify candidate modules during BRD, confirming each other results. Integrated coexpressed hub genes of eight candidate modules with PPI networks, allowed us to identify hub–hub genes that act as central genes in both coexpression and PPI networks and may be important candidates during BRD development. In total, we identified 307 hub–hub genes in eight candidate modules, most of which were potentially involved in BRD. These genes along with other members of the eight candidate modules could be important targets in the pathogenesis of BRD for future researches. Therefore, more research is needed to validate the hub–hub genes reported in this study, especially those whose role in the immune system in response to BRD is still unclear.

## Data Availability

The original contributions presented in the study are included in the article/Supplementary Material, further inquiries can be directed to the corresponding authors.

## Abbreviations

BCV, bovine coronavirus; BHV-1, bovine herpes virus type 1; BPIV-3, bovine parainfluenza type 3 virus; BRD, bovine respiratory disease; BRSV, bovine respiratory syncytial virus; BVDV, bovine viral diarrhea virus; GEO, Gene Expression Omnibus; GO, Gene Ontology; GS, gene significance; KEGG, Kyoto Encyclopedia of Genes and Genomes; ME, module eigengene; MM, module membership; MTRs, module–trait relationships; MP, module preservation; PPI, protein–protein interaction; RSV, respiratory syncytial virus; RNA-seq, RNA-sequencing; TF, transcription factor; TOM, topological overlap matrix; WGCNA, weighted gene coexpression network analysis.
