# Corrigendum: Integrated network analysis to identify key modules and potential hub genes involved in bovine respiratory disease: a systems biology approach

**DOI:** 10.3389/fgene.2025.1572285

**Published:** 2025-02-20

**Authors:** Aliakbar Hasankhani, Abolfazl Bahrami, Negin Sheybani, Farhang Fatehi, Roxana Abadeh, Hamid Ghaem Maghami Farahani, Mohammad Reza Bahreini Behzadi, Ghazaleh Javanmard, Sadegh Isapour, Hosein Khadem, Herman W. Barkema

**Affiliations:** ^1^ Department of Animal Science, College of Agriculture and Natural Resources, University of Tehran, Karaj, Iran; ^2^ Nuclear Agriculture Research School, Nuclear Science and Technology Research Institute, Karaj, Iran; ^3^ Department of Animal and Poultry Science, College of Aburaihan, University of Tehran, Tehran, Iran; ^4^ Department of Animal Science, Science and Research Branch, Islamic Azad University, Tehran, Iran; ^5^ Department of Animal Science, Faculty of Agriculture, Yasouj University, Yasouj, Iran; ^6^ Department of Agronomy and Plant Breeding, University of Tehran, Karaj, Iran; ^7^ Department of Production Animal Health, Faculty of Veterinary Medicine, University of Calgary, Calgary, AB, Canada

**Keywords:** bovine respiratory disease, RNA-seq, weighted gene co-expression network, protein-protein interaction, hub-hub genes

In the published article, there was an error in “**Affiliation 2**”. Instead of “Biomedical Center for Systems Biology Science Munich, Ludwig-Maximilians-University, Munich, Germany”, it should be “Nuclear Agriculture Research School, Nuclear Science and Technology Research Institute, Karaj, Iran”.

The authors apologize for this error and state that this does not change the scientific conclusions of the article in any way. The original article has been updated.

