# Supplementary material for: Integrated Network Analysis to Identify Key Modules and Potential Hub Genes Involved in Bovine Respiratory Disease: A Systems Biology Approach
Source: Front Genet. 2021 Oct 18;12:753839. doi: 10.3389/fgene.2021.753839 (PMC8559434; doi:10.3389/fgene.2021.753839)
Supplement: Supplementary file 3 [file Presentation1.pptx]

## Slide 1
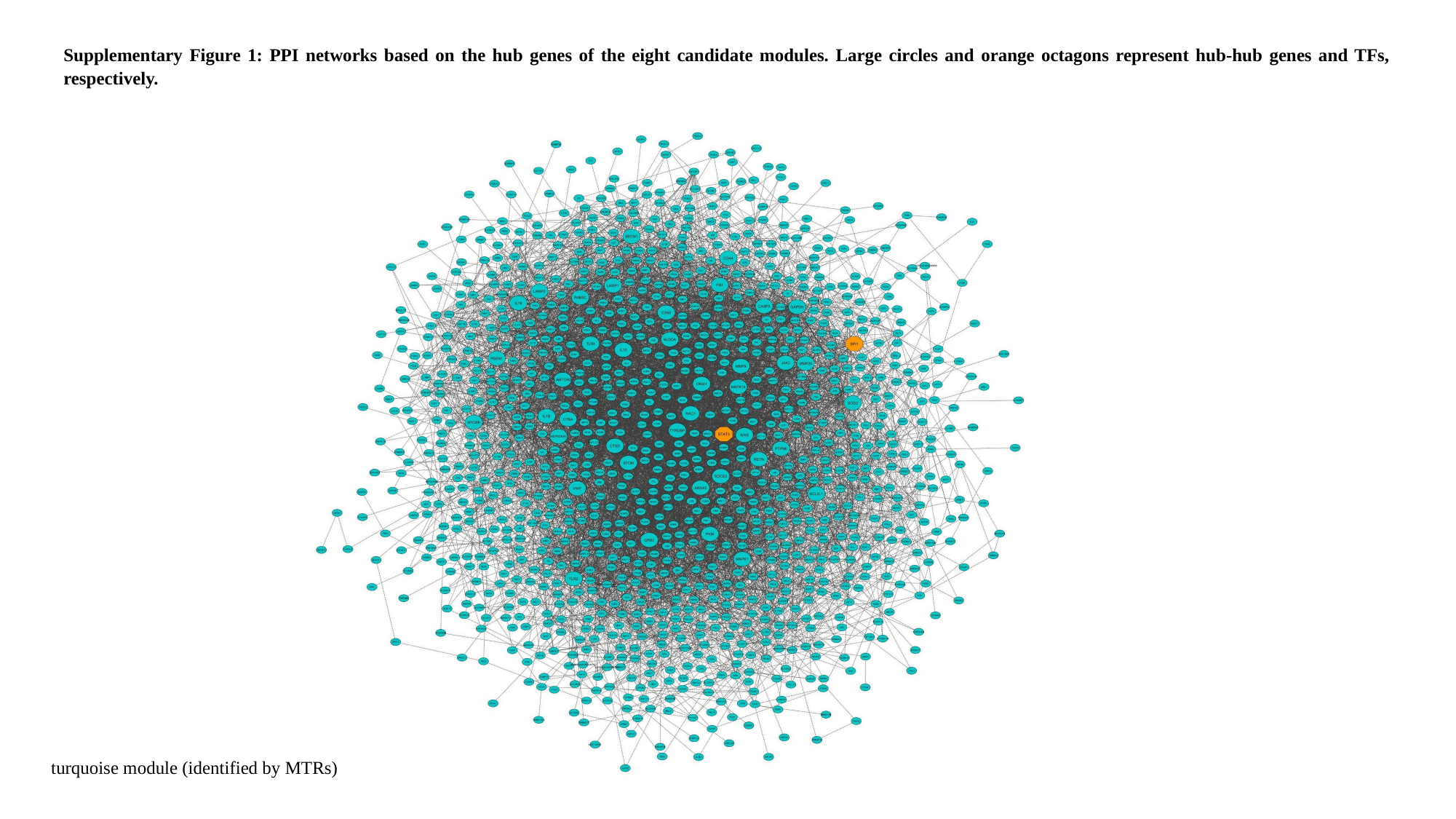

Supplementary Figure 1: PPI networks based on the hub genes of the eight candidate modules. Large circles and orange octagons represent hub-hub genes and TFs, respectively.
turquoise module (identified by MTRs)

## Slide 2
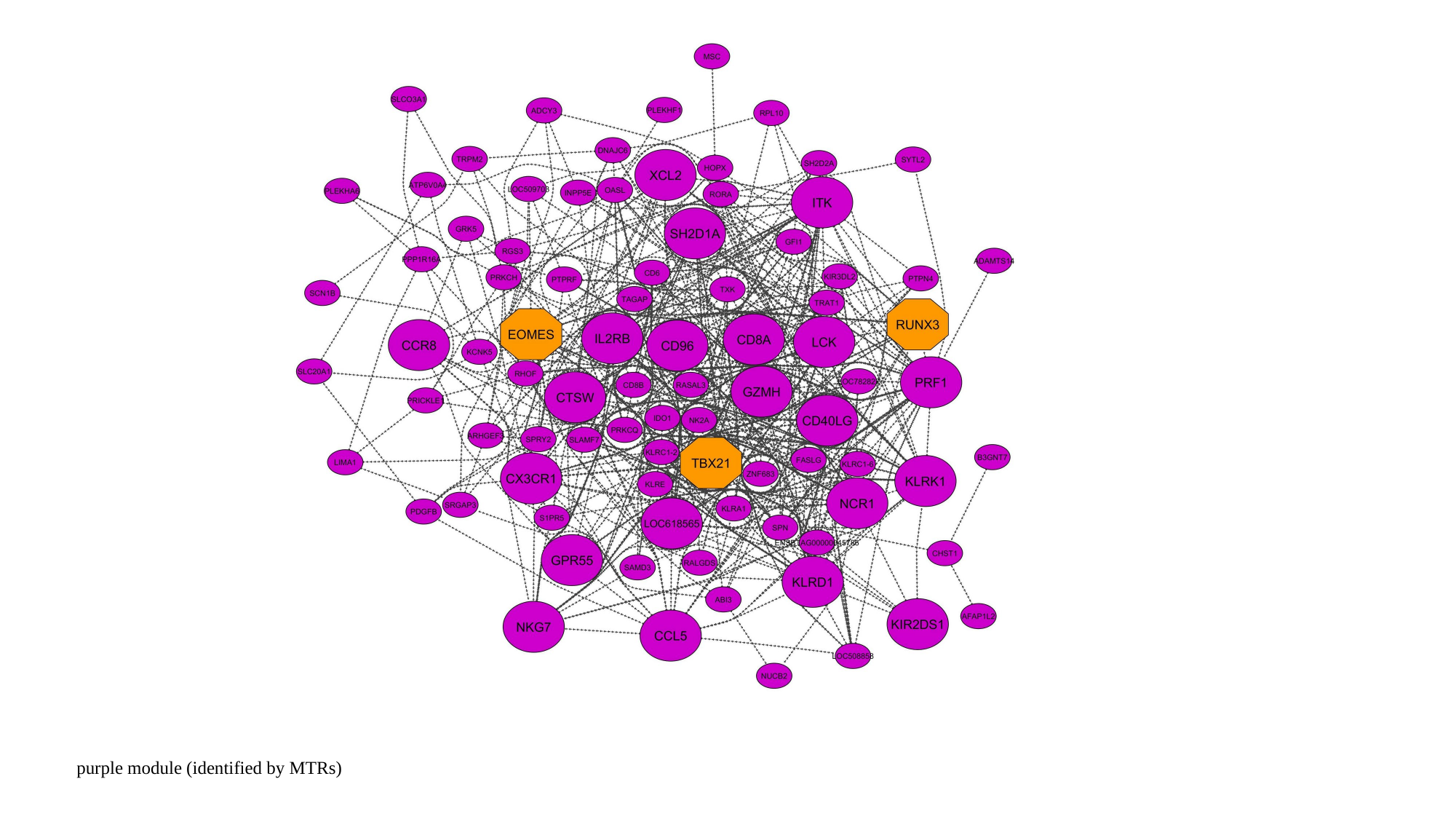

purple module (identified by MTRs)

## Slide 3
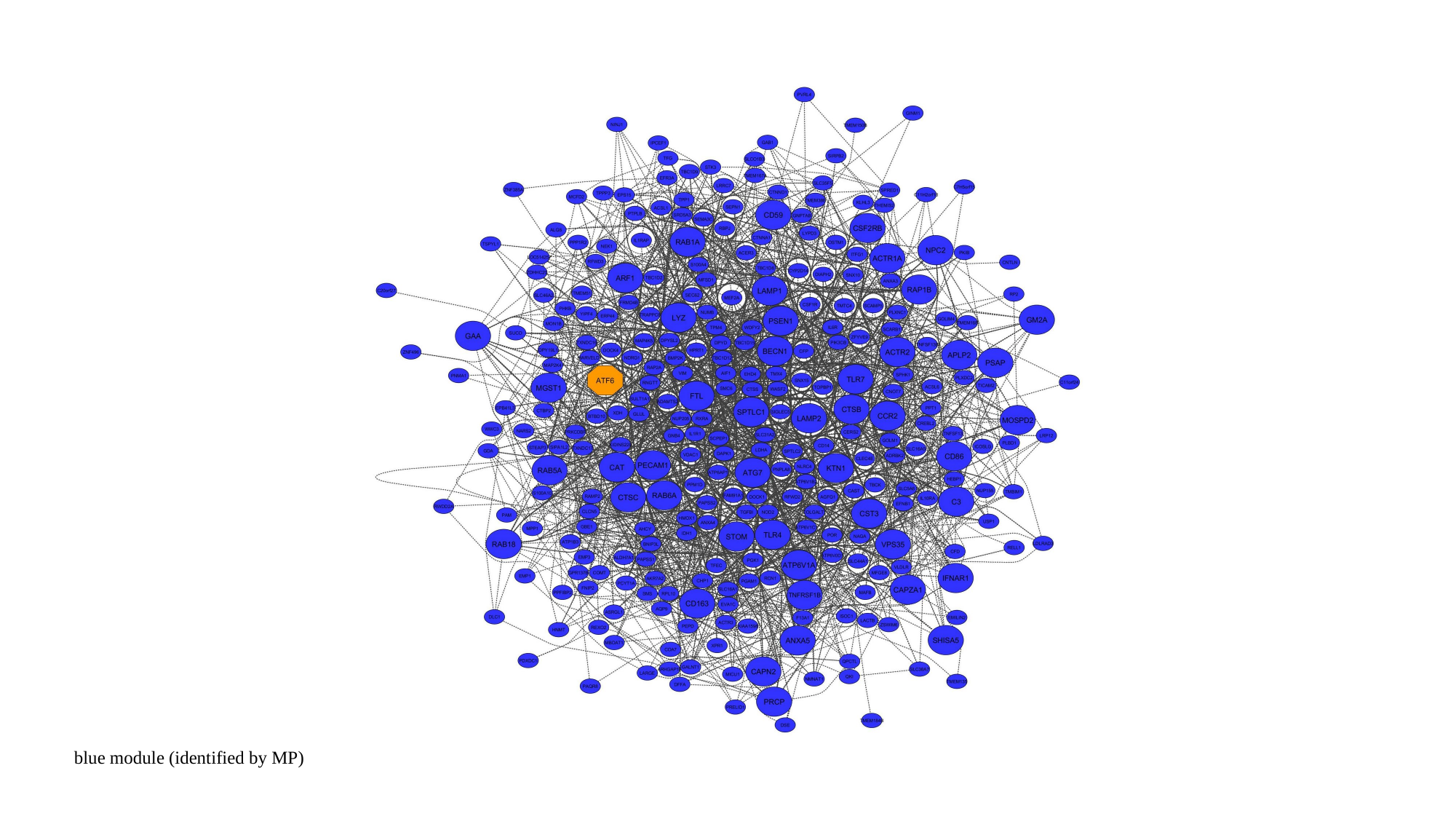

blue module (identified by MP)

## Slide 4
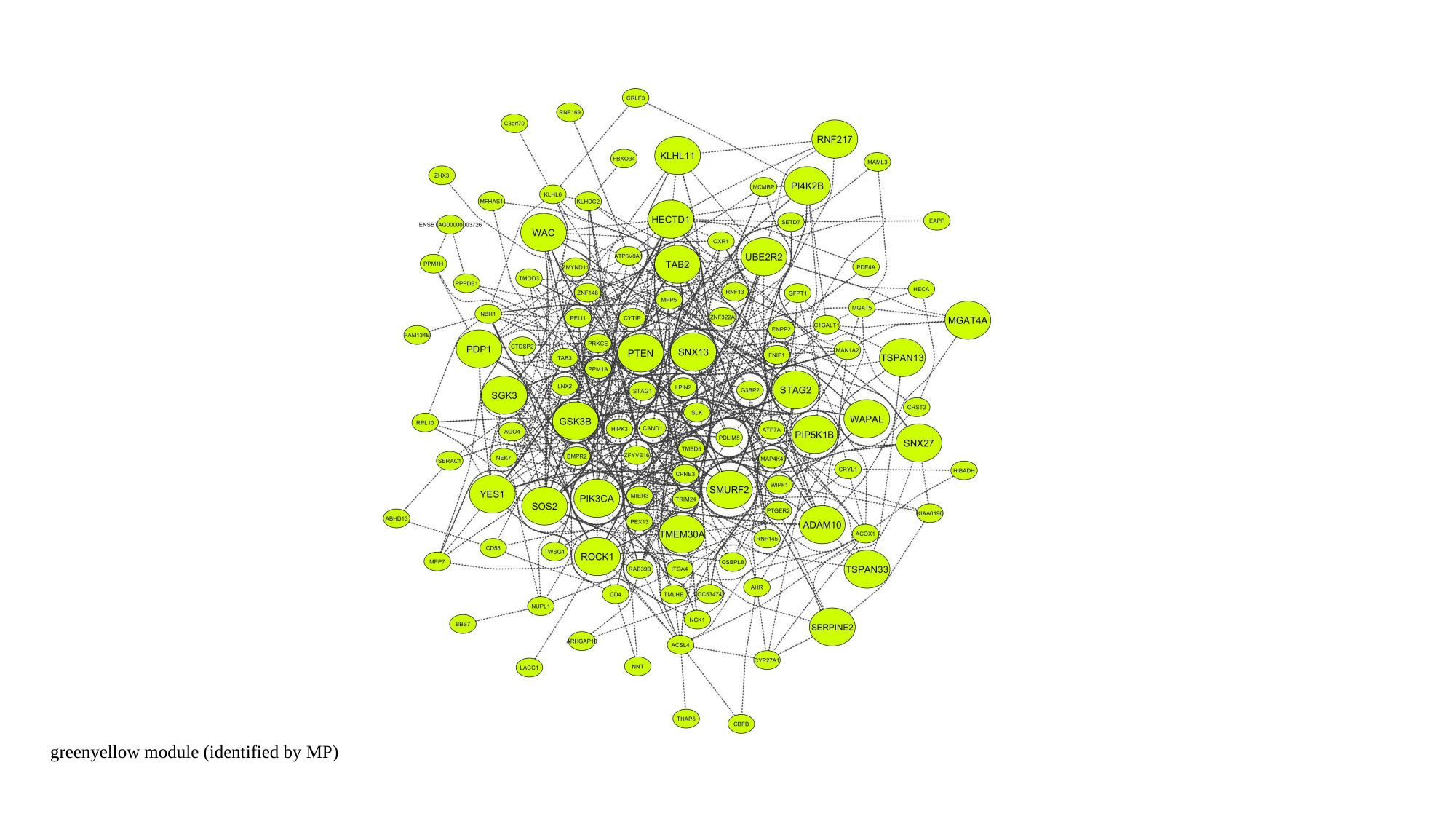

greenyellow module (identified by MP)

## Slide 5
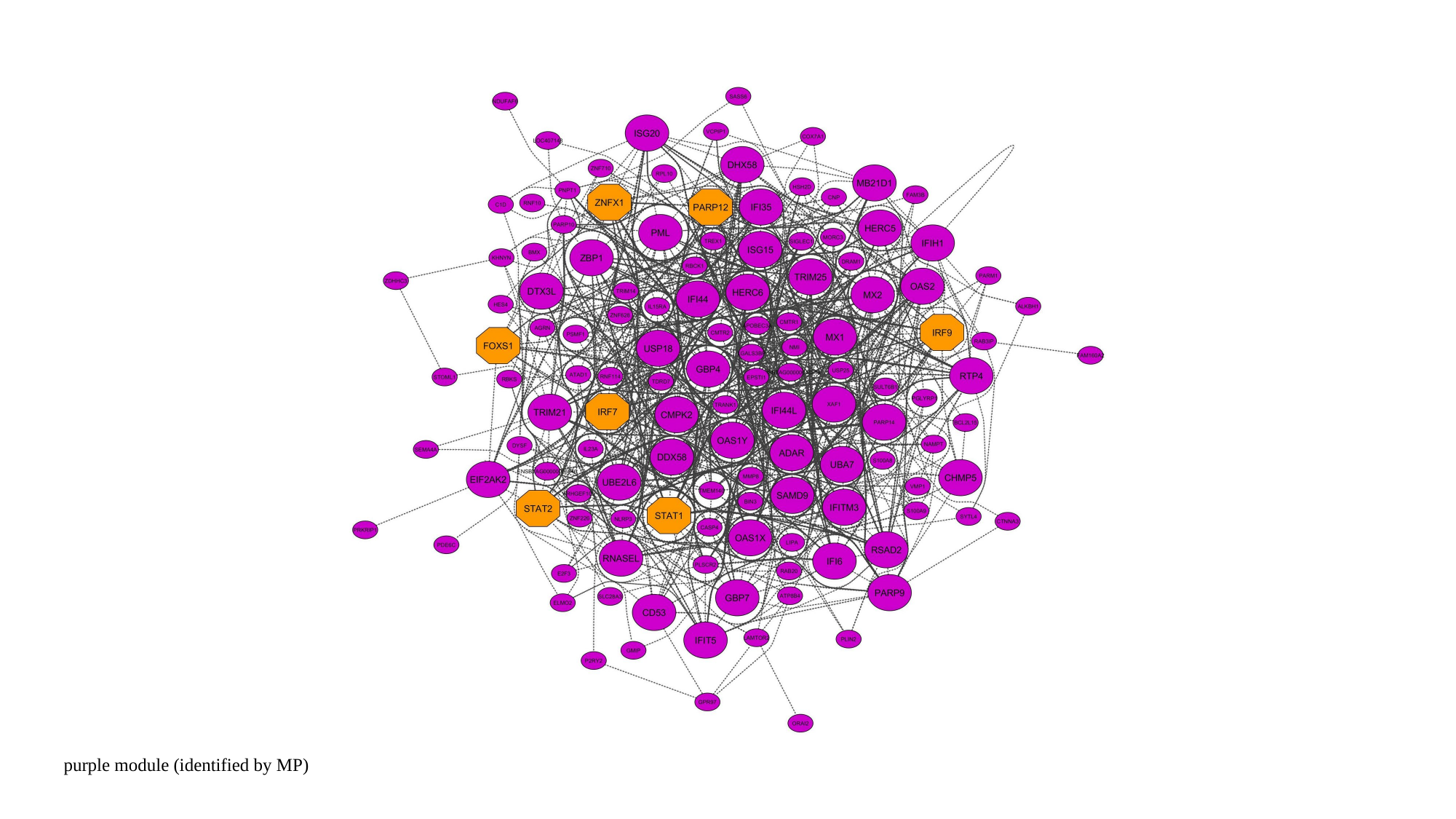

purple module (identified by MP)

## Slide 6
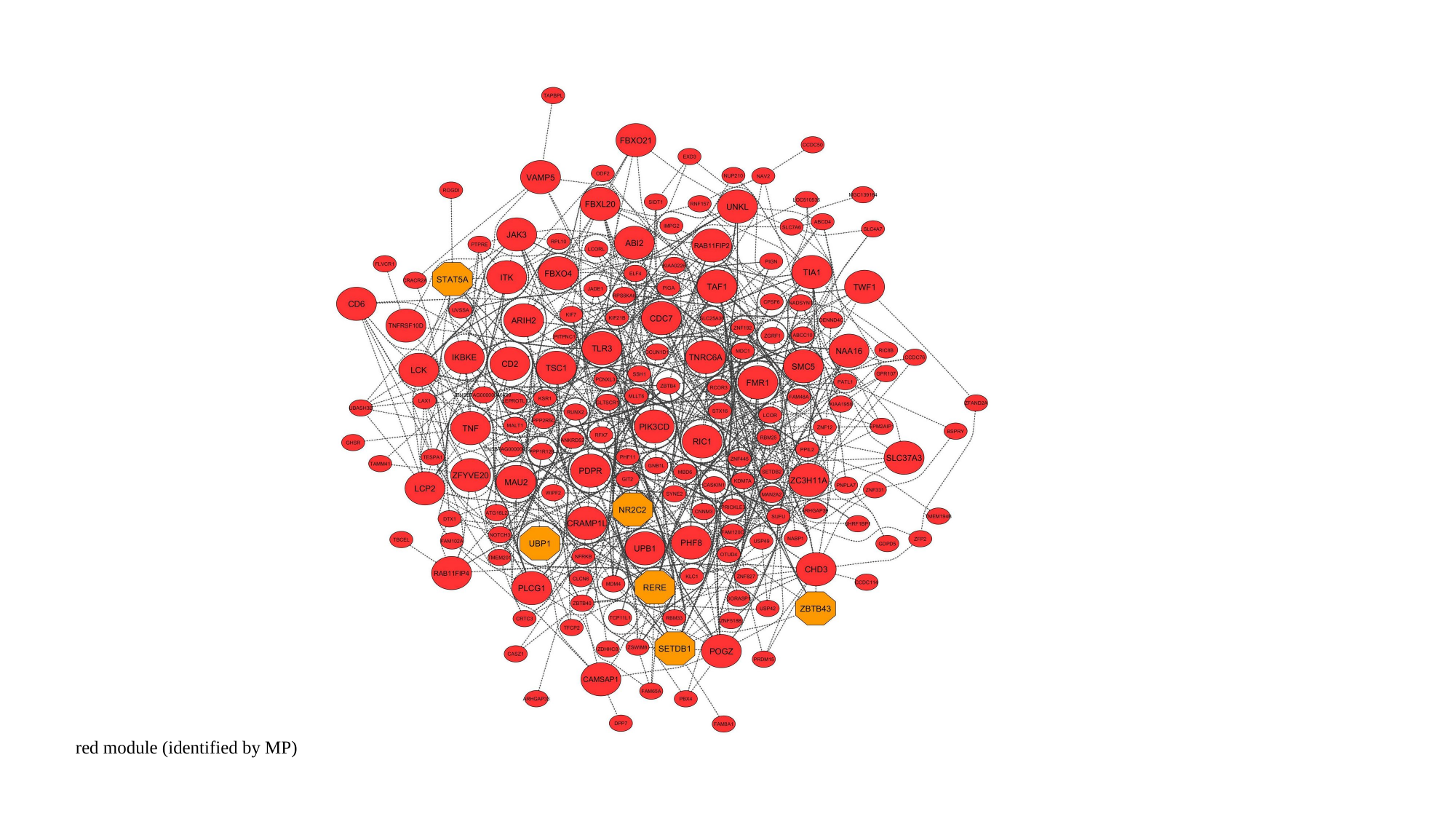

red module (identified by MP)

## Slide 7
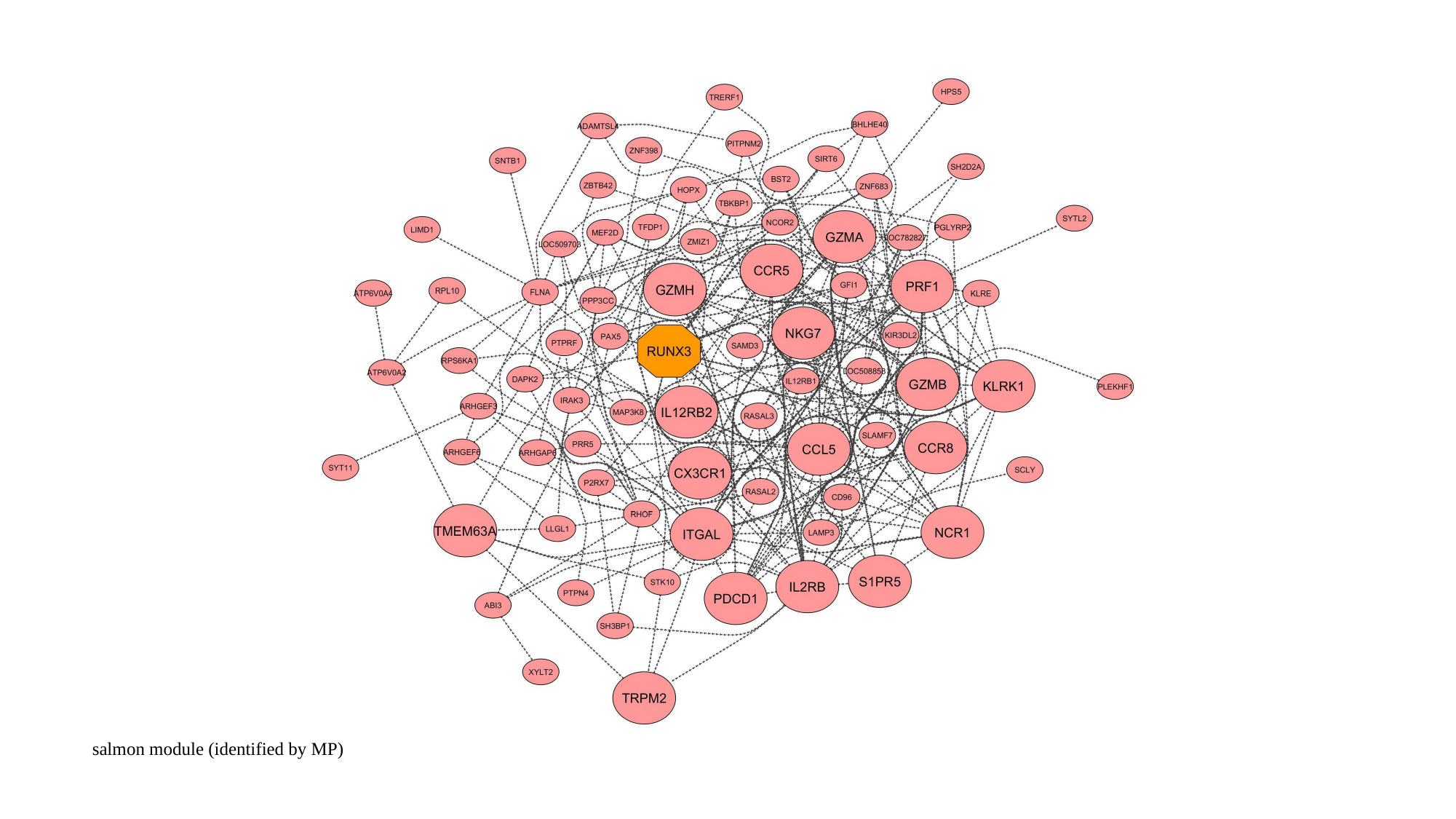

salmon module (identified by MP)

## Slide 8
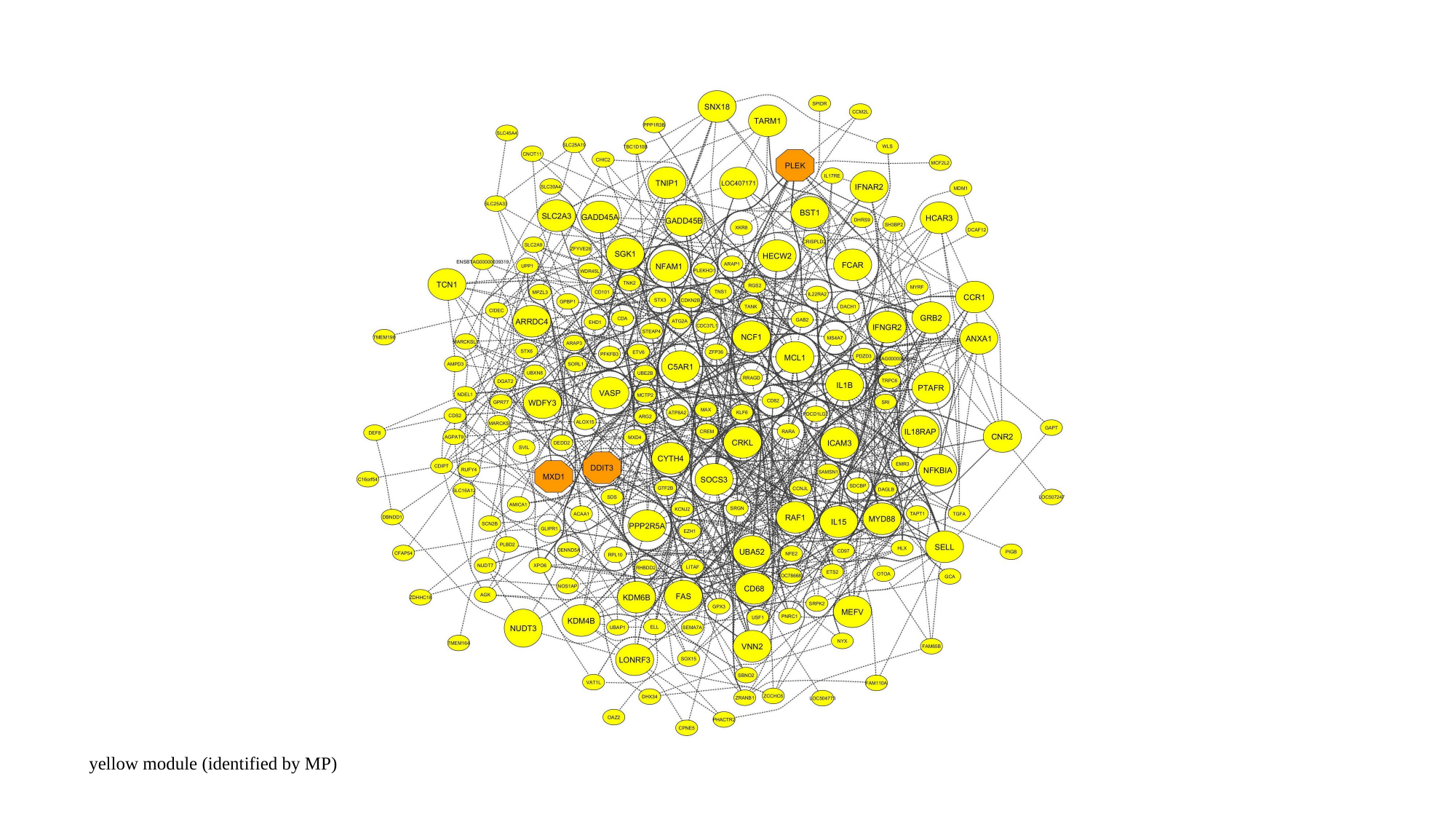

yellow module (identified by MP)
